# Associations of the MCM6-rs3754686 proxy for milk intake in Mediterranean and American populations with cardiovascular biomarkers, disease and mortality: Mendelian randomization

**DOI:** 10.1038/srep33188

**Published:** 2016-09-14

**Authors:** Caren E. Smith, Oscar Coltell, Jose V. Sorlí, Ramón Estruch, Miguel Ángel Martínez-González, Jordi Salas-Salvadó, Montserrat Fitó, Fernando Arós, Hassan S. Dashti, Chao Q. Lai, Leticia Miró, Lluís Serra-Majem, Enrique Gómez-Gracia, Miquel Fiol, Emilio Ros, Stella Aslibekyan, Bertha Hidalgo, Marian L. Neuhouser, Chongzhi Di, Katherine L. Tucker, Donna K. Arnett, José M. Ordovás, Dolores Corella

**Affiliations:** 1 Nutrition and Genomics Laboratory, Jean Mayer USDA Human Nutrition Research Center on Aging, Tufts University, Boston, MA, USA; 2 Department of Computer Languages and Systems, School of Technology and Experimental Sciences. University Jaume I, Castellón, Spain; 3 CIBER Fisiopatología de la Obesidad y Nutrición, Instituto de Salud Carlos III, Madrid, Spain; 4 Department of Preventive Medicine and Public Health, School of Medicine, University of Valencia, Valencia, Spain; 5 Department of Internal Medicine, Hospital Clinic, IDIBAPS, Barcelona, Spain; 6 Department of Preventive Medicine and Public Health, University of Navarra-Navarra Institute for Health Research (IdisNa), Pamplona, Navarra, Spain; 7 Human Nutrition Unit, Biochemistry and Biotechnology Department, IISPV, University Rovira i Virgili, Reus, Spain; 8 Cardiovascular Epidemiology Unit, Municipal Institut for Medical Research (IMIM), Barcelona, Spain; 9 Department of Cardiology, Hospital Txagorritxu, Vitoria, Spain; 10 Department of Family Medicine, Research Unit. Distrito Sanitario Atención Primaria Sevilla, Spain; 11 Research Institute of Biomedical and Health Sciences, University of Las Palmas de Gran Canaria, Las Palmas de Gran Canaria, Spain; 12 Department of Epidemiology, School of Medicine, University of Malaga, Malaga, Spain; 13 Palma Institute of Health Research (IdISPa). Hospital Son Espases. Palma de Mallorca, Spain; 14 Lipid Clinic, Endocrinology and Nutrition Service, Institut d’Investigacions Biomèdiques August Pi Sunyer (IDIBAPS), Hospital Clinic, Barcelona, Spain; 15 Department of Epidemiology, School of Public Health, University of Alabama, Birmingham, Alabama, USA; 16 Fred Hutchinson Cancer Research Center, Seattle, Washington, USA; 17 Department of Clinical Laboratory & Nutritional Sciences, University of Massachusetts Lowell, Massachusetts, USA; 18 Department of Epidemiology and Population Genetics, Centro Nacional Investigación Cardiovasculares (CNIC), Madrid, Spain; 19 Instituto Madrileño de Estudios Avanzados en Alimentación, Madrid, Spain

## Abstract

Controversy persists on the association between dairy products, especially milk, and cardiovascular diseases (CVD). Genetic proxies may improve dairy intake estimations, and clarify diet-disease relationships through Mendelian randomization. We meta-analytically (n ≤ 20,089) evaluated associations between a lactase persistence (LP) SNP, the minichromosome maintenance complex component 6 (MCM6)-rs3754686C>T (nonpersistence>persistence), dairy intake, and CVD biomarkers in American (Hispanics, African-American and Whites) and Mediterranean populations. Moreover, we analyzed longitudinal associations with milk, CVD and mortality in PREDIMED), a randomized Mediterranean diet (MedDiet) intervention trial (n = 7185). The MCM6-rs3754686/MCM6-rs309180 (as proxy), LP-allele (T) was strongly associated with higher milk intake, but inconsistently associated with glucose and lipids, and not associated with CVD or total mortality in the whole population. Heterogeneity analyses suggested some sex-specific associations. The T-allele was associated with higher CVD and mortality risk in women but not in men (P-sex interaction:0.005 and 0.032, respectively), mainly in the MedDiet group. However, milk intake was not associated with CVD biomarkers, CVD or mortality either generally or in sub-groups. Although MCM6-rs3754686 is a good milk intake proxy in these populations, attributing its associations with CVD and mortality in Mediterranean women to milk is unwarranted, as other factors limiting the assumption of causality in Mendelian randomization may exist.

Nutritional biomarkers can provide an objective assessment method for dietary exposure. Of particular interest are the genetic biomarkers of food intake, that is, single nucleotide polymorphisms (SNPs) that may function as proxies (instrumental variables) for food consumption, while reducing the confounding and measurement error^1^. Mendelian randomization involving genetic biomarkers is currently used to improve causal inferences from observational data^2^,^3^. Thus, genetic variants are considered analogously as random assignment in a clinical trial^4^. Interest is growing in the use of genetic biomarkers of dairy intake, as dairy foods have been variably associated with cardiovascular disease (CVD) and risk factors^5^,^6^,^7^. A recent Mendelian randomization study in Danes concluded that a genetic proxy [the rs4988235 in the minichromosome maintenance complex component 6 (*MCM6*)] for milk intake in northern European populations was not associated with CVD risk^8^, but more studies in other countries with varying ethnicities are needed.

Although the principles underlying Mendelian randomization are straightforward, its interpretation can be complex^9^,^10^. Selection of the appropriate proxy SNP for dairy intake may depend on the specific study populations. Lactase persistence, which confers a continued activity of the enzyme lactase in adulthood, is largely determined by genotypes of the *MCM6* gene (chromosome 2), adjacent to the gene encoding lactase (*LCT*) and influencing differential transcriptional activation of the *LCT* promoter^11^. While the *MCM6*-rs4988235 SNP (commonly called *LCT* -13910 C/T) at intron 13, is highly correlated with lactase persistence and milk intake in northern European populations, its association with dairy intake is not universal^12^,^13^,^14^,^15^. Other SNPs, including *MCM6-r*s3754686 (intron 15), occur more frequently in some global regions^11^ and represent plausible alternatives in diverse cohorts. In the Mediterranean Spanish population, where the prevalence of lactase persistence is lower than in the north of Europe, we did not find, in previous work undertaken in the PREDIMED (Prevención con Dieta Mediterránea)-Valencia study^12^, a significant association between the *MCM6*-rs4988235 SNP and dairy/milk intake^12^. Thus, after testing other SNP candidates^11^, we selected the *MCM6*-rs3754686 for analysis in the whole PREDIMED population based on our results of its better performance [trait associations, calling rate, Hardy-Weinberg equilibrium (HWE)].

Moreover, whereas evidence has accumulated regarding sex-specific differences in CVD incidence and risk factors^16^,^17^,^18^, as well as some sex-specific effects of several SNPs for CVD^19^,^20^, the implications of sex differences in Mendelian randomization studies have received less attention. Analysis of these sex-specific differences is needed as it may help us to strengthen causal inference, as previously reported^21^. Furthermore, sex differences in dairy product consumption have been reported^22^ and lactose intolerance, as subjectively experienced, also varies by sex, being higher in women^23^. Finally, another important aspect to consider in Mendelian randomization studies for outcomes as complex as CVD or mortality, is the dietary context (e.g., dietary pattern) in which the food of interest is consumed^24^. Thus, we have shown that Mediterranean Diet (MedDiet) can modulate the genetic effect on CVD risk^25^.

Therefore, in the current study we evaluated several hypotheses. First, we hypothesized that the *MCM6*-rs3754686 SNP would be associated with dairy, mainly milk, intake in Mediterranean and American populations, and that differences by sex would be observed. We further hypothesized that sex would modulate the SNP associations with CVD risk factors, CVD incidence and mortality. Therefore, we analyzed combined and sex-specific associations between this SNP and dairy intake in American (three US cohorts) and Mediterranean populations using a meta-analytic approach. We also examined the SNP associations with CVD biomarkers in all the populations, as well as with CVD and total mortality in PREDIMED. This randomized, controlled trial allowed us to further analyze the influence of the dietary context on the SNP associations with CVD and mortality.

## Results

**Figure 1:**
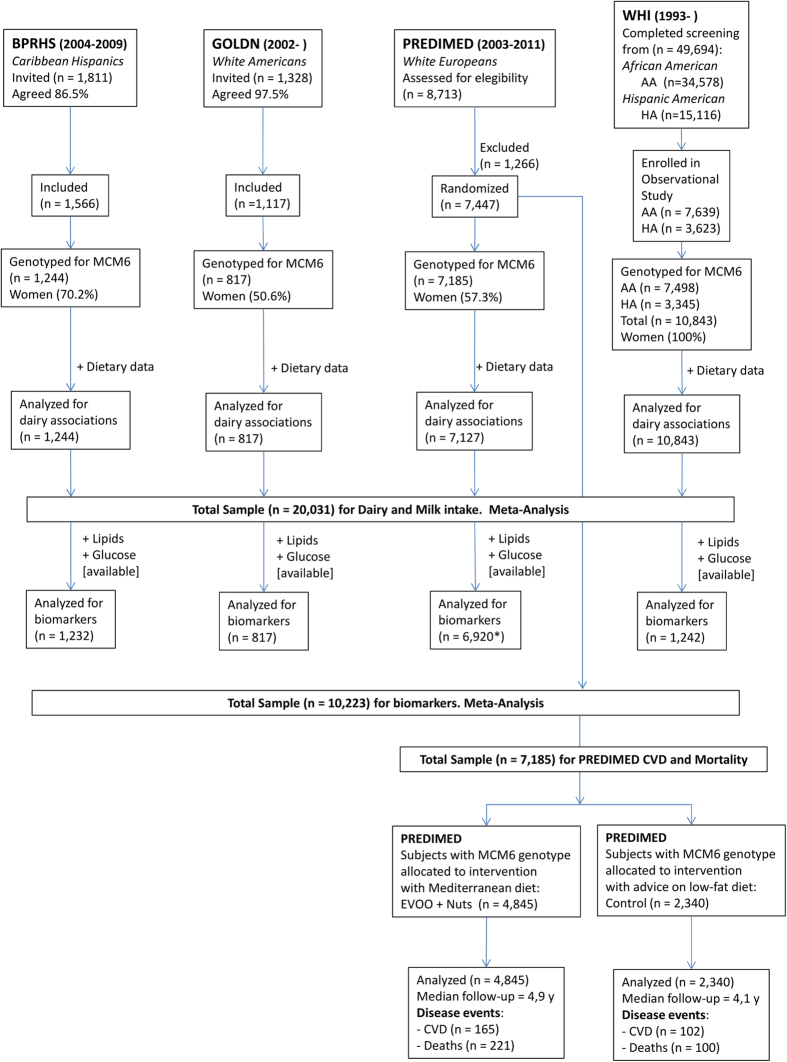
Flow-chart in BPRHS, GOLDN, PREDIMED and WHI studies. We meta-analyzed four populations including n = 20,031 subjects for the associations between the MCM6- rs3754686 polymorphism and dairy intake. N = 10,223 for the associations with CVD biomarkers, and n = 7,185 for the associations between the proxy for milk intake and incidence of CVD and total mortality.

Table 1 shows the characteristics of the populations included: Boston Puerto Rican Health Study (BPRHS), Genetics of Lipid-Lowering Drugs and Diet Network (GOLDN), Prevención con Dieta Mediterránea Study (PREDIMED) and Women’s Health Initiative (WHI) studies (n = 20,089 participants) by sex or race at baseline. Figure 1 presents the Flow Chart of the studied populations. The MCM6-rs3754686 C>T SNP is located at intron 15. It was in partial LD (D’: 0.91 and r^2^: 0.56 in PREDIMED) with the classic *MCM6*-rs4988235 C>T SNP at intron 13 (Figure S1). The *MCM6*-rs3754686 C>T SNP was genotyped in PREDIMED and imputed in the BPRHS. In GOLDN and the WHI studies, the *MCM6*-rs309180 SNP, located at intron 11, in very high LD (D’: 1 and R^2^: 0.95 in GOLDN and D’: 1 and R^2^ > 0.96 in WHI) with the *MCM6*-rs3754686 SNP, was genotyped and analyzed as a proxy. Figure S1 (Panel B) shows location, distance and LD parameters for the *MCM6* SNPs in different populations. Prevalence of the CC [lactase non-persistent genotype] was highest in WHI African Americans (56%), followed by participants in the BPRHS (36%), WHI Hispanic Americans (32%) and PREDIMED participants (23%). The lowest prevalence was detected in GOLDN participants (7%). We considered a P-value < 0.017 as statistically significant taking into account the correction for multiple comparisons (see Methods). Table S1 shows the distribution of some potentially confounding factors (age, BMI, height, sex, smoking, drinking and diabetes) across genotypes in the studied populations. We observed that these potentially confounding factors are equally distributed across genotypes for GOLDN, BPRHS, and PREDIMED (except for diabetes). In Hispanic-American women, significant differences for diabetes were detected. To discard confusion, multivariable analyses were adjusted for them.

Descriptives of the types of milk consumed as defined and computed by each cohort are shown in Table S2. In general, whole milk describes full-fat milk, reduced fat milk refers to 1% and 2% milk and skim milk refers to <1% milk.

### Association between the *MCM6*-rs3754686 SNP and dairy intake

Table 2 presents the *MCM6*-rs3754686 SNP associations with dietary intake in the four cohorts for the combined analysis (men + women or races) in every population. The *MCM6*-rs3754686 SNP was a good proxy for dairy intake, mainly total milk intake, based on the statistical significance of the associations. We show the means, SE and P-values for the untransformed variables, and have also indicated the corresponding P-values for the square root transformed variables for dairy in parentheses (see Methods). Similar P-values for both variables have been obtained, but for significance we will refer to the P-values obtained with the square root transformation. We have found nominally significant associations with total dairy and milk in every population [BPRHS (P = 0.031 for dairy and P = 0.037 for milk), GOLDN (P = (2.8 × 10^−3^) for dairy and P = 1.1 × 10^−3^ for milk), PREDIMED (P = 1.9 × 10^−6^ for dairy and P = 4.0 × 10^−6^ for milk) and WHI (P = 3.4 × 10^−66^ for dairy and P = 1.2 × 10^−65^ for milk] despite differences in allele frequency and amount of milk consumed. Moreover, additional significant associations were seen in African American (P = 4.3 × 10^−34^ for dairy and P = 1.2 × 10^−24^ for milk) and Hispanic American women (P = 2.3 × 10^−10^ for dairy and P = 4.5 × 10^−11^ for milk) (Table S3). In terms of determination coefficients (R^2^) and F values, the associations with milk were stronger in Hispanic-American (R^2^: 2.8%; F: 298.1) and African-American women (R^2^: 2.12%; F: 152.7) followed by the other populations: PREDIMED (R^2^: 0.4%; F: 24.8), GOLDN (R^2^: 1.5%; F: 5.8) and BPRHS (R^2^: 0.7%; F: 3.9).

Interestingly, the *MCM6*-rs3754686 SNP presented a dosage effect, with higher intakes in individuals homozygous for the lactase persistence allele (T). Thus, regression coefficients per variant T-allele (additive model) were estimated and all the populations were combined in a random-effects meta-analysis (Figure S2). Strong associations were found for total dairy (**A**) and milk (**B**) with regression coefficients of 30.3 (95% CI 21.4–39.3) g/d (P < 0.001) and 26.4 (95% CI 16.7–36.2) g/d (P < 0.001) per variant-T allele, respectively. A small but significant association was detected for yogurt (**C**), with regression coefficient of 2.52 (95% CI 1.18–3.86) g/d (P < 0.001) per T-allele and no association for cheese (**D**) (regression coefficient of 0.29; 95% CI: −0.09–0.67) g/d (P = 0.051).

Considering that the MCM6-rs3754686 SNP in the African American women of WHI study was in slight HWE disequilibrium, we undertook sensitivity analysis by carrying out the meta-analysis without the AA-WHI women. These meta-analyses associations (presented in Figure S2 as P’ estimations) were similar although of a lesser magnitude.

**Figure 2:**
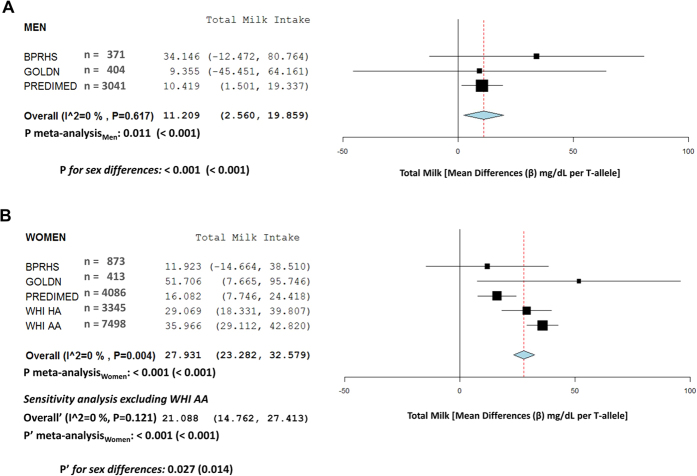
Meta-analysis of the association between the *MCM6*-rs3754686 polymorphism and total milk intake according to sex in BPRHS, GOLDN, PREDIMED and WHI studies. Forest plots: (**A**) total milk in men, and (**B**) total milk in women, show adjusted regression coefficients and 95% CI (expressed in g/d and estimated per one copy of the T-allele; LCT genotypes coded as 0, 1 and 2 according to the number of T-alleles) for the corresponding intake in each study. The diamond shows the meta-analyzed associations in a fixed-effects model. The I^2^ statistic was calculated for heterogeneity. P_meta-analysis_ indicates the P-value obtained in the meta-analysis including all populations. P’_meta-analysis_ indicates the P-value for the meta-analysis obtained in the sensitivity analysis excluding the WHI AA women. In both cases, results for raw data and square-root transformed data (values in parentheses) for milk are presented. P and P’ for sex differences indicate the P-values for heterogeneity by sex in the total (P) and the sensitivity (P’) meta-analysis.

In addition to the combined analysis, we examined the MCM6-rs3754686 associations with dairy intake stratified by sex (Tables S3 and S4). We observed that the SNP associations with total dairy and milk intake tended to be higher in women (Table S3) than in men (Table S4). We also carried out a sex-specific meta-analysis for milk intake (Fig. 2) and estimated sex-specific association results (fixed effects) and computed the P-value for sex differences in the meta-analyzed beta coefficients as previously described^26^. Thus, the increase in milk intake per each T allele in women was 27.9 (95% CI 23.3–32.6) g/d versus 11.2 (95% CI 2.6–19.9) g/d in men and the P-value for sex differences in the meta-analysis including all populations, was statistically significant (P < 0.001). A sensitivity analysis excluding African-American WHI women also found statistically significant results (Fig. 2) and reduced the heterogeneity in women. Thus, although the magnitude and statistical significance for sex-specific differences in milk intake per T-allele were attenuated in this meta-analysis excluding African Americans, the differences in the meta-analyzed regression coefficients by sex still reached the statistical significance (P’ = 0.014) in the fixed effect meta-analysis supporting sex differences in the magnitude of the association between the SNP and milk intake. Taking into account the heterogeneity (P for I^2^) observed in women including African Americans, an additional random-effect meta-analysis for women (analyzing all populations) was undertaken (results not shown). With this meta-analyzed beta for women, we computed sex-specific differences with men, and also obtained a statistically significant P-value for differences in the square root transformed milk intake estimations (P = 0.013). Similar results were found for total dairy (Figure S3) in the sex-specific meta-analyses including all the populations as well as after exclusion of African American WHI women in the sensitivity analysis.

**Figure 3:**
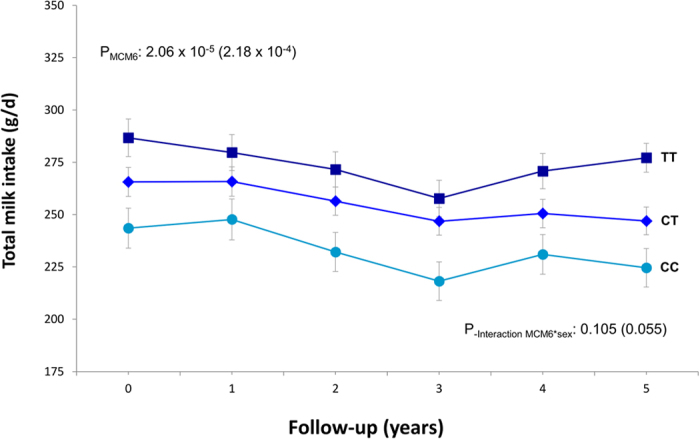
Longitudinal effect of the *MCM6*-rs3754686 polymorphism on total milk intake over a 5-y follow-up period in the PREDIMED study in men and women combined. Adjusted means of milk intake are expressed in g/d yearly depending on the genotype in all subjects having data for all the measurements (n = 2087). Error bars indicate the standard error of means. P-values for the overall effect of the polymorphism as well as the P-values for the interaction term between the MCM6 SNP and sex, were estimated from a repeated-measures ANOVA model adjusted for sex, age, field center, diabetes, smoking, drinking, and total energy intake. The P-values without parentheses refer to the untransformed continuous variables, whereas values in parentheses refer to square-root transformed variables for milk.

In the PREDIMED study, we were, moreover, able to analyze the association of the MCM6-rs3754686 SNP with total dairy and milk intake measured at baseline and up to 5 years (n = 2087 subjects with complete data at baseline and yearly). In the men and women combined analysis, we observed that the SNP was significantly associated with dairy (Figure S4) and milk intake (Fig. 3) throughout the period. The interaction by sex was borderline significant for milk (P = 0.055). When we analyzed the effects separately in men and women (not shown), we found that for women the longitudinal association of the SNP with dairy intake tended to be more significant (P < 0.001) than in men (P = 0.049).

### Associations between the *MCM6*-rs3754686 proxy for milk intake and fasting glucose and lipids

**Figure 4:**
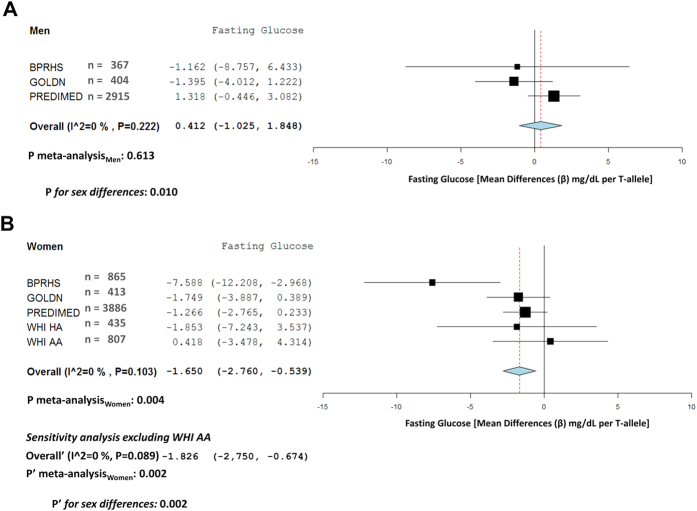
Meta-analysis of the association between the *MCM6-*rs3754686 polymorphism and fasting glucose according to sex in BPRHS, GOLDN, PREDIMED and WHI studies. Forest plots: (**A**) men, (**B**) women, show adjusted regression coefficients and 95% CI (expressed in mg/dL and estimated per one copy of the T-allele; LCT genotypes coded as 0, 1, and 2 according to the number of T-alleles) for the corresponding intake in each study. The diamond shows the meta-analyzed associations in a fixed-effects model. The I^2^ statistic was calculated for heterogeneity. P_meta-analysis_ indicates the P-value obtained in the meta-analysis including all populations. P’_meta-analysis_ indicates the P-value for the meta-analysis obtained in the sensitivity analysis excluding the WHI AA women. P and P’ for sex differences indicate the P-values for heterogeneity by sex in the total (P) and the sensitivity (P’) meta-analysis.

Table 3 shows associations between *MCM6*-rs3754686 and fasting glucose and lipids. These biomarkers were available in nearly all BPRHS, GOLDN or PREDIMED, but only available in a subsample of women participating in the WHI study. Although some nominally significant associations with certain lipids were observed in specific populations, no strongly replicated association was noted for the lipid traits. In the analysis by sex (Tables S5 and S6 for men and women, respectively), we detected more consistent results for glucose in women. The meta-analysis of these results (Fig. 4) showed that the SNP was significantly associated with a lower fasting glucose concentration in women (−1.65; 95% CI: −2.76, −0.54 mg/dL per T-allele), but not in men (0.41; 95% CI: −1.03, 1.85 mg/dL). The differences between the meta-analyzed regression coefficients between men and women were statistically significant (P = 0.010). These differences were also observed when the African-American WHI women (although in HWE in the biomarkers sub-sample) were removed from the meta-analysis in the sensitivity analysis (P = 0.002). Models were further adjusted for milk intake and no significant changes in the coefficients were observed. This may indicate that either the SNP effects are independent from milk consumption, or could reflect a high measurement error in that the milk intake, such that variable has a high measurement error and the adjustment for this imprecisely badly measured variable did not alter the beta coefficient.

### Associations between milk intake and fasting glucose and lipids

Associations between observationally measured milk intake and glucose and lipid outcomes were analyzed combined and stratified by sex in each population. No consistent associations were found (Table S7) and no statistically significant heterogeneity by sex was observed. In order to compare these results with the previous ones we meta-analyzed the sex-specific estimations of the association between observed milk intake and fasting glucose but did not observe any association either in men or women, or any heterogeneity per sex (Figure S5).

### Associations between the *MCM6-*rs3754686 SNP and CVD incidence and total mortality in the PREDIMED study

The association analyses with CVD incidence and total mortality could only be undertaken in the PREDIMED study. Over a median of 4.8 years of follow-up, 267 new cases of CVD were detected. The *MCM6-*rs3754686 SNP was not significantly associated with CVD incidence in the whole population [HR (Hazard Ratios): 1.02, 95% Confidence interval (CI): 0.86–1, 22; P = 0.785 per variant T-allele in the multivariable adjusted Model 2; See Methods]. Details of the incidence rates and HR per genotype are provided in Table 4. For total mortality, 322 deaths were confirmed. Likewise, no significant associations between the *MCM6-*rs3754686 proxy for milk intake and total mortality were found (HR: 1.07, 95% CI: 0.92–1.26; P = 0.378) in the multivariable adjusted Model 2. Additional adjustment for milk intake or macronutrients did not change the statistical significance of the associations.

**Figure 5:**
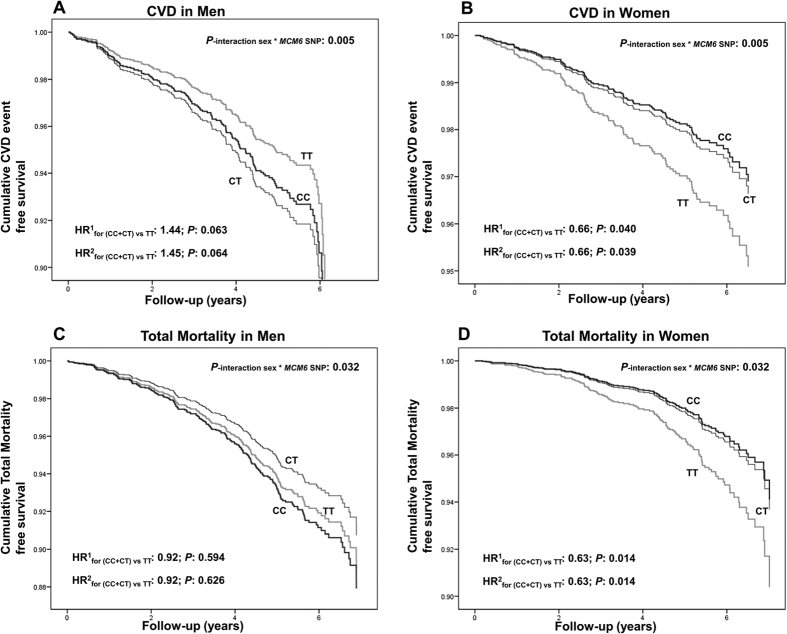
Kaplan Meier curves of cumulative CVD-free survival or mortality free survival depending on the *MCM6*-rs3754686 polymorphism and sex in the PREDIMED participants. N = 3065 men and n = 4120 women were analyzed: (**A**) CVD incidence in men, (**B**) CVD incidence in women, (**C**) total mortality in men and (**D**), total mortality in women. Multivariable Cox regression models with outcome of CVD incidence or total mortality were fitted as indicated in methods. HR and 95% CI were obtained in the multivariable adjusted models: HR^1^: Model 1 (adjusted for sex, age, field center and dietary intervention group) and HR^2^: Model 2 (adjusted for variables in model 1 plus BMI, diabetes, drinking, smoking, physical activity, medication (hypertension, dyslipemia and glucose) and total energy intake at baseline). For these estimations, as well for the interaction terms, a recessive model was computed. P for interactions between the MCM6 SNP and sex in the corresponding multivariable adjusted Cox regression model were P = 0.005 for CVD, and P = 0.032 for total mortality in Model 2.

When the heterogeneity was analyzed per sex, we obtained statistically significant interaction terms between the *MCM6*-rs3754686 SNP and sex in determining CVD incidence (P = 0.005) and total mortality (P = 0.032). The former exceeded the cutoff-value for multiple comparisons, and the latter exceeded the nominal value, supporting the stratified analysis (P < 0.1 for predefined groups)^27^. Details of the incidence rates and HR per *MCM6* genotypes for CVD incidence and total mortality in men and women are provided in Tables S8 and S9, respectively. Likewise, Fig. 5 shows event-free survival Kaplan Meier curves for CVD in men (**A**), and CVD in women (**B**), total mortality in men (**C**) and total mortality in women (**D**) depending on the *MCM6-*rs3754686 SNP. Taking the noted recessive effect into account, carriers of the lactase non-persistence allele (C) were grouped and compared with homozygous individuals for the lactase persistence allele (T). In men no significant associations were detected. Conversely, in women, we found significant associations with CVD incidence (at P < 0.05) and mortality (at P < 0.017). Thus, the lactase non-persistence allele (CC + CT genotypes) was associated with lower CVD incidence (HR: 0.66; P = 0.039 in model 2) and total mortality (HR: 0.63; P = 0.014 in model 2) in comparison with the TT genotype (homozygous for lactase persistence). The statistical significance of these associations was not changed after further adjustments for milk intake (Model 3) or total fat and carbohydrates (Model 4).

### Modulation of the effects of the associations between the MCM6-rs3754686 SNP and CVD incidence and total mortality by dietary intervention in the PREDIMED study

We explored whether intervention with MedDiet modulated the associations between the SNP and CVD or mortality. No modification of the effect was detected in men either for CVD incidence or total mortality (P for interactions >0.1). However, in women we detected a P-value for interaction (P = 0.09) between the SNP and the MedDiet intervention in determining CVD, as well as total mortality (P-interaction = 0.09). These P-values (<0.1) are suggestive of heterogeneity^27^. Then we carried out the stratified analyses of the associations for CVD in the MedDiet (Table S10) and control groups (Table S11) as well as for total mortality in the MedDiet (Table S12) and control groups (Table S13). Figure S6 shows cumulative event-free survival Kaplan Meier curves for CVD in women in the MedDiet (**A**), CVD in women in the control group (**B**), total mortality in women in the MedDiet (**C**) and total mortality in women in the control group (**D**) by *MCM6*-rs3754686 SNP. No associations were found for women in the control group (HR: 1.03; P = 0.983 for CVD and HR: 0.97; P = 0.928 for total mortality). However, an increased protective effect of lactase non-persistence allele against CVD incidence and total mortality was found for women in the MedDiet intervention group. In the recessive model, carriers of the non-persistence allele (C) had lower incidence of CVD in comparison with those homozygous for the LP allele (HR for CC+CT versus TT: 0.54; P = 0.013 in model 2). A similar protective effect of the non-persistence allele was observed for total mortality in women (HR: 0.57; P = 0.013 in model 2). Additional adjustment for milk intake did not change the statistical significance of the associations.

### Effects of the measured milk intake on CVD incidence and total mortality in the PREDIMED study

When we specifically analyzed milk intake by tertiles (proximate tertiles: <200 g/d (less than one glass/d), 200 g/d (one glass/d) or >200 g/d (more than one glass/d)) or dichotomously (<200 g/d or > = 200 g/d), we did not observe any significant association between milk intake and these events in the combined analysis or in men or women (Table S14). When milk intake was high (>200 g/d) no increased risk of CVD or total mortality was found neither in the whole population nor in men or women (P-interaction milk*sex >0.1). No additional heterogeneity by the MedDiet intervention was found. Figure S7 shows in women cumulative event-free survival Kaplan Meier curves by milk intake [2 categories based on the median (200 g/d) were considered] for: (**A**) CVD in the MedDiet group; (**B**) CVD in the control group; (**C**) total mortality in the MedDiet group and (**D**) total mortality in the control group. Similar results were found in men (not shown).

## Discussion

We confirmed that the *MCM6*-rs3754686 C>T SNP (in very high LD with the *MCM6*-rs309180 SNP), previously identified as a widely distributed marker of lactose tolerance^11^, is a good proxy for milk intake in Southern European (Mediterranean from Spain) and American populations (including African Americans, Hispanic Americans, US Puerto Ricans and Whites), the T-allele being associated with higher dairy intake, especially with milk. The results of our meta-analysis also suggest a greater association of the T-allele with milk intake in women, but this heterogeneity by sex needs to be confirmed in other populations in order to better assess its importance. On using the *MCM6*-rs3754686 C>T SNP as an instrument for assessing the causal association between milk intake and CVD biomarkers (glucose and lipids) through Mendelian randomization, we did not find any significant association (having taken correction for multiple comparisons into account) with any of these biomarkers in the population as a whole (men and women). However, on undertaking sex-stratified analysis, we found the *MCM6*-rs3754686 C>T SNP to be significantly associated with fasting glucose in the meta-analysis of women, but not in men, the differences between them being statistically significant. Likewise, on analyzing the association between the *MCM6*-rs3754686 C>T SNP and incidence of CVD and total mortality in the PREDIMED dietary intervention, we did not obtain any association in the population as a whole, but the analysis per previously defined dietary and sex sub-groups did allow us to identify statistically significant associations.

As far as we know, this is the first time that a meta-analysis in multi-ethic populations has shown a strong association of the *MCM6*-rs3754686 C>T SNP with total dairy intake, mainly milk. This marker may be more appropriate as an instrumental variable of milk intake in Mediterranean and American populations than the *MCM6*-rs4988235, originating in Northern Europe^11^, as there are previous studies that have found that the *MCM6*-rs4988235 does not predict milk intake in all populations^12^,^13^,^14^,^15^. We also show for the first time a longitudinal association between the *MCM6*-rs3754686 SNP and dairy intake, specifically milk intake, over a 5-year follow-up period. Moreover, in contrast to other studies on the *MCM6*-rs4988235^8^,^28^ in Danes in which a dominant effect is reported, the association of the *MCM6*-rs3754686 SNP appears to reflect an additive model. Although we observed the association between the *MCM6*-rs3754686 SNP and milk intake to be statistically significant on studying men and women jointly, we wished to also investigate the associations in men and women separately given that previous studies had not provided these data in detail^8^,^28^ and that prior evidence exists for sex-based heterogeneity both in milk intake^22^ and CVD risk^17^,^18^,^29^,^30^. The results of the sex-differences in meta-analysis suggested that the effect of the association between the SNP and milk intake is greater in women than in men and reaches statistical significance (including after correction for multiple comparisons) when all populations are included. However, the statistical significance of the difference by sex was attenuated on eliminating African-American women in the sensitivity analysis (as there was a departure from HWE in this group). This departure from the equilibrium, in addition to a potential bias^31^, may simply represent a slight departure due to the large sample size of this population (>7000 individuals for dairy intake analysis), taking into account that for biomarkers (only available for 807 women, the HWE was reached; P > 0.05), or that for evolutionary reasons, the equilibrium is still being reached. Bergholdt *et al*.^8^ proposed a similar argument in their Mendelian randomization study between the *MCM6*-rs4988235 SNP in Danes, where they observed deviations from the HWE in two Danish populations and they did not exclude these populations from the Danes analysis. In our study, we undertook a sensitivity analysis for all the associations, excluding the African-American women for comparative purposes. Although our elimination of a large group attenuated both magnitude and statistical significance of effects, there is still a significant difference in the association of the proxy SNP with milk intake between men and women, which should be the subject of further studies.

Previous studies have employed the sex-stratified approach for examining heterogeneity by sex in Mendelian randomization studies^21^,^32^,^33^,^34^,^35^, for alcohol, rather than milk consumption. When the ALDH2 association with alcohol consumption was analyzed by sex, higher associations were observed in men than in women^21^,^34^,^35^ thus providing prior evidence of heterogeneity by sex for instrumental variables. Social and cultural factors influence food intake and may contribute to sex-specific findings for milk as well as alcohol; for example, women may traditionally be more likely to prepare meals, the lactose content of which may be consistent with their own LP status.

On analyzing the association of the instrumental variable with CVD risk factors, we did not find any statistically significant association in the population as a whole. However, in the sex-stratified meta-analysis, the T-allele was significantly associated with lower fasting glucose in women, but not in men, the heterogeneity by sex being statistically significant (P for sex differences <0.017). The association in women, where the T-allele that is associated with higher milk intake is inversely associated with fasting glucose, is consistent with previous studies that show that the greater intake of milk or dairy products is associated with a lower risk of type 2 diabetes^36^,^37^. Nevertheless, in observational studies, heterogeneity by sex has not been investigated and warrants further investigation. We have previously identified sex as an important source of heterogeneity in genetic association studies, including CVD and related phenotypes (obesity, insulin resistance and diabetes)^16^, also including milk^38^.

Similarly for CVD risk factors, when we analyzed the association of the MCM6 SNP proxy for milk intake in the PREDIMED study with incidence of CVD and total mortality in the population as a whole, we did not find significant associations. This would support previous studies that reported no association between milk intake and CVD or mortality^39^,^40^,^41^ and also the Mendelian randomization results of no association of the MCM6 SNP with CVD in Danes^8^. However, on examining heterogeneity by sex, we did find significant differences in the association of the MCM6 SNP between men and women: in women the T-allele associated with higher milk intake is associated with higher CVD incidence and mortality, primarily in the group of women that received the MedDiet intervention. Previous studies have also suggested a possible interaction by sex in the effects of milk intake on mortality^42^,^43^, but more evidence needs to be gathered. We know of no previous study analyzing the association between the *MCM6* locus and total mortality available for comparison with our results. Sex-specific findings associated with the *MCM6* locus in Mendelian randomization studies have not previously been described, and such findings have important implications for its potential use as a proxy for intake. The mechanisms by which the CC genotype (lactase nonpersistence) is protective against CVD in women in the current study are not clear due to the ambiguous relationships of the *MCM6*-rs3754686 SNP with glucose and lipids. Furthermore, the complexity of our observations is mirrored in the Mendelian randomization in Danes^28^ that reported an interaction between milk intake and the lactase persistence genotype for the outcome of diabetes, in such a way that lactase persistence was protective against diabetes in milk consumers and tended to increase risk in non-milk consumers. In other words, the effect of the genotype depended directly on the intakes of the participants, complicating the reliance on the genotype as a determinant of dietary intake that can predict disease. This observation is analogous to our findings that lactase non-persistence status reduces CVD risk in the context of the Mediterranean diet intervention, a dietary pattern that is characterized by relatively low milk intake^44^. The authors of the study on Danes argued that their observation could be due to a collider stratification bias, consisting of unmeasured confounding induced by selection bias^45^. In our PREDIMED study we cannot fully assess whether our observation is due to a collider bias or not, as the risk of that bias always increases when stratification is undertaken, but, in the case of the PREDIMED study, the strata were assigned at random, thus minimizing the bias that lifestyle variables may cause aside from the dietary intervention itself. Therefore our results suggest that *MCM6* SNPs may have several limitations when used as genetic proxies to assess the role of milk or dairy products in complex diseases if no additional considerations (i.e. dietary pattern) are taken into account. Of note is the limitation that the MCM6 SNP does not provide information of the specific type of dairy product (e.g., low-fat, high-fat) consumed. These limitations have been outlined as relevant^4^,^46^.

In interpreting our observations several areas are amenable to hypotheses. While the positive selection signal at the lactase locus is among the strongest of any in the human genome^47^,^48^, natural selection is likely to have modified many additional loci, in a fashion referred to by McCullough *et al*. as a “domino effect”, whereby a change at one locus was linked to a series of additional genomic adaptations^49^. Some authors have reported that lactase persistence is associated with a variety of diverse disease phenotypes, including Crohn’s disease and common cancers^50^,^51^. Hypothetically, we could postulate that lactase persistence was associated with selection for a heightened immune response, which may be manifested in some as an overall pro-inflammatory phenotype that could be linked to a range of modern, chronic diseases. Therefore, pleiotropy, which refers to a single locus affecting multiple traits^4^,^10^, may be present in the associations observed. In contrast, CVD protection conferred by the MedDiet is postulated to act via anti-inflammatory, anti-oxidative mechanisms^52^,^53^. We may extend this reasoning to suggest that a combination of protections stemming from female sex, an anti-inflammatory dietary intervention (Mediterranean) and lower genetically-based inflammatory burden (lactose non-persistence genotype) may have enabled us to detect the specific signal in the current study. Clearly, additional studies directed at investigation of potential links between lactase persistence and inflammation/oxidative stress are needed.

In conclusion, the current study represents an extensive exploration of a single *MCM6* SNP for associations with dairy intake and disease phenotypes, which is strengthened by its use of meta-analysis in several populations and longitudinal, intervention-based outcomes. Our findings revealed that the *MCM6*-rs3754686 is associated with milk intake in Mediterranean and American populations and add new information about context-specific effects in Mendelian randomization studies. The potential heterogeneity by sex or the dietary context should be examined in further studies, and if confirmed, the corresponding interaction terms may be used as instrumental variables to strengthen the causality level of the Mendelian randomization approach.

## Methods

We analyzed 20,089 subjects from the following populations (detailed data in Fig. 1 and Table S15):

### Boston Puerto Rican Health Study (BPRHS)

The BPRHS is a five-year longitudinal cohort study of nutrition, health and aging, within one of ten NIH-funded Centers on Population Health and Health Disparities. Study design and methods have been previously described^54^. Detailed characteristics of the study participants are shown in Fig. 1 and Table S15. Here we included 1244 individuals (873 women and 371 men), aged 45–75 years, self-identified Puerto Ricans living in urban Massachusetts, USA. The institutional review boards at Tufts University, Northeastern University and the University of Massachusetts at Lowell approved the study protocol and all participants provided informed consent. The methods were carried out in accordance with the approved guidelines.

### Genetics of Lipid Lowering Drugs and Diet Network (GOLDN) Study

We analyzed 817 individuals (413 women and 404 men, mean age 49 years) of European ancestry from the GOLDN study who were re-recruited from the National Institutes of Health National Heart, Lung and Blood Institute Family Heart Study (Table S15). Field centers were located in Minneapolis, Minnesota and Salt Lake City, Utah, USA. The study details and related methodology of GOLDN have been described^55^. The protocol was approved by the Institutional Review Board at the Universities of Alabama, Minnesota, Utah, and Tufts, and all participants provided written informed consent. The methods were carried out in accordance with the approved guidelines.

### Prevención con Dieta Mediterránea (PREDIMED) trial

The PREDIMED (www.predimed.es) is a multi-center, randomized, controlled clinical trial (controlled-trials.com number, ISRCTN35739639) aimed at assessing the effects of the Mediterranean Diet (MedDiet) on the primary prevention of CVD^44^. The total number of randomized participants was 7,447 (Fig. 1 and Table S15). The 7,185 participants (4120 women and 3065 men) included with genotype data available did not differ in the main characteristics from those of the total cohort. Eligible participants were community-dwelling persons (55–80 years for men; 60–80 years for women) who fulfilled at least one of two criteria: type-2 diabetes or 3 or more cardiovascular risk factors^44^. Participants were randomly assigned to: a MedDiet (two groups, one supplemented with extra-virgin olive oil, the other with nuts), and a control group (low-fat diet). Median follow-up time was 4.8 years. The Institutional Review Board of each participating center approved the study protocol, and all participants provided written informed consent. The methods were carried out in accordance with the approved guidelines.

### Women’s Health Initiative (WHI) Study

The WHI is a study of risk factors for cardiovascular disease, cancer, osteoporosis and other chronic diseases of postmenopausal women (Fig. 1, Table S15). WHI included a clinical trial (CT) component and an observational study (OS). Eligible women could be randomized into one, two, or all three of the CTs components. Women were eligible for WHI participation if they were 50–79 years of age at screening and postmenopausal^56^. For this analysis we included participants from a WHI genetic study: the SNP Health Associated Resource (SHARe) cohort. SHARe included 12,007 minority women (n = 8,405 African Americans and n = 3,602 Hispanic). Eligible women included those that self-reported African-American or Hispanic race/ethnicity, who participated either in one of the CTs or the OS, and who signed a separate informed consent permitting use of their DNA for use in genome-wide scans [African American (n = 7498) and Hispanic American (n = 3345)]. Baseline biomarkers for WHI were obtained for the current study from the National Center for Biotechnology Information (NCBI) Database of Genotypes and Phenotypes (dbGaP). The Institutional Review Board of each participating center and the independent WHI Data and Safety Monitoring Board approved the study protocol and all participants provided informed consent. The methods were carried out in accordance with the approved guidelines.

### Biochemical methods for glucose and lipids

In GOLDN, fasting glucose was measured using a hexokinase-mediated reaction on the Hitachi commercial kit (Roche Diagnostics). Triglycerides were measured by a glycerol-blanked enzymatic method on the Roche COBAS FARA centrifugal analyzer. Total cholesterol and high density lipoprotein cholesterol (HDL-C) were measured on the Hitachi 911 Automatic Analyzer (Roche Diagnostics) using a cholesterol esterase/cholesterol oxidase reaction and low density lipoprotein cholesterol (LDL-C) was measured directly. Methods have been previously described^55^. In the BPRHS^54^, the Olympus Au400e with Olympus glucose reagents (Olympus America Inc.) was used to measure fasting glucose. Fasting triglycerides and HDL-C were measured with Olympus HDL-C reagents (OSR6195) and Olympus triglyceride reagents (OSR6033). In PREDIMED fasting glucose, total cholesterol, triglycerides and HDL-C were measured using standard enzymatic methods as previously described^56^. In participants whose triglyceride concentration was <400 mg/dL, LDL-C concentrations were estimated using the Friedewald formula. In the WHI, serum glucose was measured using a hexokinase method, total cholesterol and triglycerides were measured enzymatically, HDL-C was measured using manganese sulfate precipitation and LDL-C was calculated using the Friedewald equation. Details have been previously described^57^ and provided in Table S15.

### Outcome ascertainment (CVD incidence and mortality)

In the PREDIMED study, the primary endpoint was the occurrence of the first major CVD event and comprised stroke, myocardial infarction or cardiovascular death^44^. We also assessed total mortality. We used four sources of information to identify end-points: (1) direct participant contact; (2) family physicians; (3) yearly review of medical records; and (4) linkage to the National Death Index. Medical records of deceased participants were requested. The End-point Adjudication Committee adjudicated the cause of the death and confirmed cardiovascular events. End-points confirmed by the committee occurring between October 1, 2003, and December 1, 2010 were included in the analyses. The criteria for adjudicating outcomes are detailed elsewhere^44^.

### Dietary Intake

Validated food frequency questionnaires (FFQ) were used in each population to estimate dietary intakes. In the BPRHS, the National Cancer Institute-Block FFQ of 126 items was revised, and validated in this population^58^. In GOLDN, the Diet History Questionnaire (DHQ), consisting of 124 food items and developed by the National Cancer Institute, was used. Validity of the DHQ has been assessed^59^. In PREDIMED, food consumption was determined by a 137-item validated FFQ^60^. In WHI, the baseline FFQ was developed specifically for WHI and included regional foods and foods consumed in minority populations. The Nutrition Data Systems for Research (NDS-R) was used to estimate average daily nutrient intake over the previous 3-mo period and included 122 foods or food groups^61^. For all cohorts, derived food groups representing total dairy, milk (all types), cheese and yogurt, as defined by each cohort, were used in analyses.

### Genotyping

For both the GOLDN study and the WHI SNP Health Association Resource (SHARe) study, genotypes were obtained using the Genome-Wide Human SNP Array 6.0 (Affymetrix, Santa Clara, CA, USA, www.affymetrix.com). In the BPRHS, the Affymetrix’s Axiom Genome-Wide LAT Array, which was designed especially for Hispanic populations and contains probe sets to genotype 817,810 SNPs, were used to conduct the genome-wide genotyping. In PREDIMED genotyping was carried out in a 7900HT Sequence Detection System (Life Technology, Foster City, CA, USA) using a fluorescent allelic discrimination TaqMan TM assay. In PREDIMED the *MCM6*-rs3754686 was determined by TaqMan assays and in the BPRHS the *MCM6*-rs3754686 was imputed and evaluated. For imputation, the 1000 genome project genotypes were used with reference haplotype panels from the Nov.23 2010 release of the 1000 Genomes project using a MaCH-Admix (http://www.unc.edu/~yunmli/MaCH-Admix/) by Yun Li (University of North Carolina of Chapel Hill). Genotypes for imputation were obtained using the Affymetrix’s Axiom Genome-Wide LAT Array (717,275 autosomal SNPs genotyped). These data were selected to create the input file for MaCH-Admix (see Table S15 for details). In WHI and GOLDN, the proxy SNP *MCM6*-rs309180, with a high LD with the *MCM6*-rs3754686 (D’ = 1 and r^2^ = 0.95), was selected for genotyping. Genotypes for this SNP were obtained using the arrays above mentioned. Genotype frequencies for the *MCM6*-rs3754686 or *MCM6*-rs309180 SNPS, did not deviate from Hardy-Weinberg equilibrium expectations in BPRHS (P = 0.08), GOLDN (P = 0.42), PREDIMED (P = 0.39) or WHI Hispanic Americans (P = 0.67), but deviated (at P < 0.05) in WHI African Americans in the large sample for dairy intake associations (P = 7.8 × 10^−6^).

### Statistical analysis

Triglycerides were log transformed for statistical analysis and P-values for the log transformed variable were reported. Continuous variables for dairy products did not follow a normal distribution and were square root transformed to improve normality and maintain the zero values for consumption. Analyses using dairy products as the outcome were performed using both versions of the dairy variables, that is untransformed and square root transformed. However results were similar using the two models variables. In Tables and Figures for dairy intake associations, both P-values for untransformed (without parenthesis) and square root transformed variables (within parenthesis), were shown. However, in the text, only P-values were reported for the square root transformed.

Unadjusted associations between MCM6-rs3754686 SNP and dairy intake, and intermediate biomarkers (glucose and lipids) were evaluated using analysis of variance (ANOVA) techniques with an additive genetic model for the (0, 1 or 2 variant alleles). Multivariable adjusted genetic associations for dairy intakes (total dairy, milk, cheese and yogurt) and biomarkers were evaluated by linear regression/general linear models using two adjusted models, one minimally adjusted including age, sex, race and population-specific covariates [including ancestral admixture (West African, Southern European and American Indian) in the BPRHS using principal components as previously reported^62^, family structure and field center in the GOLDN Study and race/ethnicity (Hispanic and African American) in the WHI Study] and BMI, smoking, drinking, physical activity, diabetes, medication and total energy intake. For dairy intake, models for untransformed as well as for square root transformed variables were fitted and the corresponding P-values computed. Association analyses were carried out both for the whole corresponding population and stratified by sex or race as indicated. Standard regression diagnostic procedures were used to ensure the appropriateness of the regression models.

For the meta-analyses of the associations between MCM6-rs3754686 SNP and dairy intake or continuous biomarkers we applied Open MetaAnalyst, an open-source meta-analysis software that uses R as the underlying statistical engine, and Python for the graphical user interface. We applied fixed or random effects models depending on the analyzed variable and group. In the meta-analysis including men and women, we assumed heterogeneity and used the DerSimonian-Laird analysis method for continuous random-effects model with Estimate, Lower/Upper bounds of 95% CI and P-values, and the heterogeneity coefficients. For dairy intake, we meta-analyzed both untransformed and square root transformed variables. In the forest plots we showed adjusted regression coefficients and 95 CI% (per copy of the T-allele) for the untransformed variables. We showed the P-values for the meta-analysis corresponding to the untransformed variables as well as the P-values for the meta-analysis of the square root transformed variables (within parenthesis). In addition, we carried out a sensitivity analysis excluding African American women (because of the departure of the H-W equilibrium) and obtained the corresponding effect-sizes and the P-values (expressed as P’) for both the untransformed and the square root transformed variables.

We also carried out sex-stratified meta-analyses in men and women and computed the effect-size estimated and P-values for the untransformed and the square root transformed variables for total milk and total dairy intake. In the stratified analysis by sex, we first assumed no heterogeneity and carried out fixed effects meta-analysis. For both men and women we showed the P-values including all the populations as well as the P’-values for the sensitivity meta-analysis excluding African American women. We also computed P-values (P-diff) testing for difference between the corresponding meta-analyzed men-specific (β_Men_) and women-specific (β_Women_) beta-estimates and SE for milk intake, total dairy intake or fasting glucose using the t statistics as previously reported^26^:


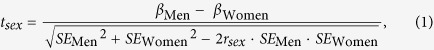


R indicates de Spearman rank correlation coefficient between men and women for each estimation and the degrees of freedom (df) for the corresponding P estimation for a given t and sample size was as follows:


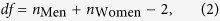


The P-values for the difference in the SNP association with milk and total dairy were estimated for the untransformed and square root transformed variables. In addition, in the meta-analysis including all the women, some heterogeneity was detected and we applied the random-effect procedure to estimate the corresponding regression coefficient and the t statistic for sex-differences was additionally calculated. For fasting glucose, only untransformed data were used and the fixed effects model was considered.

The longitudinal association between the MCM6-rs3754686 SNP and dairy and milk intake was analyzed in a repeated measures ANOVA model adjusted for covariates. Dairy consumption of dairy intake or milk were available at baseline and at 1y, 2y, 3y, 4y and 5y in n = 2078 PREDIMED participants. Men and women were pooled and we tested the interaction term between the MCM6-rs3754686 SNP and sex in the repeated measures model adjusted for potential confounders. Untransformed and square root transformed variables for dairy and milk intake were analyzed.

Moreover, we analyzed the associations of milk intake (observational) with fasting glucose and lipids in each population (BPRHS, GOLDN, PREDIMED and WHI) in its entirety and stratified by sex. Milk intake was analyzed as continuous and multivariable adjusted regression coefficients (for untransformed and square root transformed intakes) were estimated. Meta-analysis of the beta coefficients for observational milk intake in determining fasting glucose was also carried out as described above.

To examine the association between the MCM6-rs3754686 SNP and incidence of major CVD (total, stroke and myocardial infarction) and total mortality in participants of the PREDIMED study, we used Cox regression models with length of follow-up as the primary time variable. Analyses were based on the intention-to-treat principle. The exposure time was calculated as the time between randomization and the date of a major CVD, the date of the last interview, December 1st 2010, or the date at death, whichever came first as previously reported^44^. We first estimated incidence rate for the three genotypes and fitted codominant, recessive and additive models. Hazard ratios (HR) with 95% confidence intervals (CI) for the *MCM6* SNP were estimated. Models were multivariable adjusted for covariates as indicated (Model 1 and Model 2). An additional adjustment of the estimations by total milk intake was considered in Model 3 as well as the additional adjustment for total fat and carbohydrates (Model 4). Formal tests for interaction between the MCM6-rs3754686 SNP and sex were assessed in determining CVD or total mortality by analyzing the product term of these variables in the multivariable Cox regression models including the main terms and covariates. The likelihood ratio test was used to obtain the P values for interactions. As statistically significant interaction terms were obtained, we carried out stratified analyses of the associations between the MCM6-rs3754686 SNP and CVD incidence and total mortality in men and women separately. Furthermore, considering that the PREDIMED is a randomized, controlled dietary intervention trial, we evaluated the modulation by diet of the associations between the genetic proxy for milk intake and CVD and total mortality by sex. In men and women we tested the statistical significance of the interaction terms between dietary intervention and the MCM6 SNP. We considered a P < 0.1 as suggestive of interaction^27^ and estimated the HRs for the *MCM6* SNP, stratified by dietary intervention groups (MedDiet versus control diet). For the Cox regression analyses, we graphically checked the assumption of proportional hazards for the genetic factor (MCM6 SNP) by looking at the Log-Log plot of survival. We obtained parallel lines for the curves of genotypes in all the models fitted supporting the proportional hazards. In addition, Kaplan-Meier survival curves were plotted to estimate the probability of remaining free of total CVD or total mortality during follow-up.

Finally, in the PREDIMED trial we also evaluated the association between the observational milk intake (proximate tertiles and as dichotomous) and CVD incidence and total mortality in the population as a whole and by sex. Cox regression models were fitted and adjusted for covariates as indicated (Model 1 to Model 3).

In BPRHS, GOLDN and WHI, SAS (version 9.2 for Windows) was used to analyse data. In PREDIMED, statistical analyses were performed with the IBM SPSS Statistics version 21.0, NY. All tests were two-tailed and the nominal significance level was set at a P-value < 0.05. In addition, the Bonferroni correction was applied to compensate for multiple comparisons. Taking into account that most of the variables analyzed (total dairy with total milk, HDL-C with triglycerides and other cardiovascular biomarkers, etc.) have a high correlation between them, it would not be appropriate to correct the alpha value per number of comparisons undertaken, as this would increase the probability of false negatives too much. So, for the correction for multiple comparisons, we consider three groups of variables: the dairy intake group, the biomarkers group and the incidence of hard events group. In this way, the value of alpha corrected for multiple comparisons would be P < 0.05/3 = 0.017. Using this correction we minimize the possible risk of false positives without increasing too much the probability of false negatives Thus, the adjusted alpha was 0.05/3 = 0.017. The significance of each original (uncorrected) test was assessed at this level. Thus, a P-value < 0.017 was considered to be statistically significant taking into account the correction for multiple comparisons. In terms of sample size, we included 4 studies with different size and power. For the associations between the MCM6 SNP and dairy products, we analyzed 20,031 individuals (1244 from the BPRHS, 817 from GOLDN, 7127 from PREDIMED and 10843 from the WHI). According to the sample size considerations for Mendelian randomization studies suggested by Brion *et al*.^63^ taking into account two parameters: the proportion of variation in the exposure variable explained by the genetic predictor and the true causal association between the exposure and outcome variable, for associations with cardiovascular biomarkers (n = 10,223) our study (with and overall r^2^ ranging from 0.7 to 1.5% for milk) was adequately powered (power >80% and alpha 5%) to detect clinically relevant associations (established at the 10% level) and even lower, with cardiovascular biomarkers in both men and women. In the stratified analyses, power was higher in women than in men and also our sample size allowed us to detect statistically significant heterogeneity by sex with differences in the meta-analyzed beta of 2 mg/dL (for glucose) per variant allele. For associations with CVD incidence and mortality, previous sample size calculations in the PREDIMED study^44^ established that an n = 7000 with a median follow up period ~4.8 years and the estimated incidence between groups was adequately powered (>80% at alpha = 5%) to detect HR > 1.25 compared with the reference in the recessive model. In the stratified analyses by sex, the power was reduced, and, for women, HR > 1.37 would be detected as statistically significant at beta = 20%. Higher effects would be detected as statistically significant in the stratified analysis by sex and diet (HR > 1.45 in women in the MedDiet group). The power to detect multiplicative interaction terms at P < 0.017 was limited in the stratified analysis by sex and diet and P < 0.1 was considered as suggestive of heterogeneity^27^.

## Additional Information

**How to cite this article**: Smith, C. E. *et al*. Associations of the MCM6-rs3754686 proxy for milk intake in Mediterranean and American populations with cardiovascular biomarkers, disease and mortality:Mendelian randomization. *Sci. Rep.* **6**, 33188; doi: 10.1038/srep33188 (2016).

## Supplementary Material

Supplementary Information

## Figures and Tables

**Table 1:** Population Characteristics, stratified by sex or race*.

	Boston Puerto Rican Health Study (BPRHS)		P^1^	Genetics of Lipid Lowering Drugs and Diet Network (GOLDN)		P^1^	Prevención con Dieta Mediterránea (PREDIMED)		P^1^	Women’s Health Initiative (WHI)		P^1^
	Men (n = 371)	Women (n = 873)		Men (n = 404)	Women (n = 413)		Men (n = 3065)	Women (n = 4120)		African American Women (n = 7498)	Hispanic American Women (n = 3345)	
Age, yrs	56.7 ± 8.0	57.4 ± 7.7	0.164	49 ± 16	49 ± 16	0.363	66.0 ± 6.5	67.7 ± 5.8	< 0.001	61.1 ± 6.8	60.0 ± 6.6	<0.001
BMI, kg/m^2^	29.7 ± 5.1	32.7 ± 7.0	<0.001	28.6 ± 4.7	28.4 ± 6.2	0.180	29.3 ± 3.3	30.4 ± 4.1	<0.001	30.9 ± 6.3	28.8 ± 5.5	<0.001
Plasma glucose, mg/dL	122 ± 54	121 ± 54	0.617	106 ± 22	99 ± 16	0.000	124 ± 41	117 ± 41	<0.001	108 ± 36	103 ± 29	0.006
Total cholesterol, mg/dL	174 ± 43	190 ± 39	<0.001	190 ± 39	194 ± 43	0.236	201 ± 39	217 ± 39	<0.001	221 ± 43	221 ± 39	0.932
LDL-C, mg/dL	101 ± 35	112 ± 35	<0.001	124 ± 31	124 ± 35	0.093	128 ± 35	132 ± 35	<0.001	139 ± 39	132 ± 35	0.018
HDL-C, mg/dL	38.7 ± 11.6	46.4 ± 11.6	<0.001	42.6 ± 7.7	50.3 ± 15.5	<0.001	50.3 ± 11.6	58.1 ± 15.5	<0.001	58.1 ± 15.5	54.2 ± 15.5	<0.001
Triglycerides, mg/dL	177 ± 142	159 ± 106	0.115	151 ± 115	124 ± 80	<0.001	142 ± 89	133 ± 71	0.011	124 ± 62	159 ± 71	<0.001
Total energy, kcal/day	2687 ± 1321	2093 ± 1068	<0.001	2500 ± 1500	1780 ± 817	<0.001	2446 ± 623	2150 ± 560	<0.001	1598 ± 914	1660 ± 957	0.010
Total fat intake, %energy	32.2 ± 5.3	30.8 ± 5.1	<0.001	35.8 ± 6.7	35.1 ± 6.9	0.010	38.79 ± 6.8	39.59 ± 6.8	<0.001	34.8 ± 8.2	33.7 ± 8.2	<0.001
Saturated fat intake, %energy	9.8 ± 2.4	9.3 ± 2.2	1.3 × 10^−3^	12.1 ± 2.7	11.5 ± 2.6	<0.001	9.9 ± 2.2	10.0 ± 2.2	0.135	11.0 ± 3.1	10.9 ± 3.1	0.264
Carbohydrate intake, %energy	50.0 ± 7.4	52.4 ± 7.5	<0.001	47.5 ± 8.6	50.3 ± 8.1	<0.001	41.1 ± 7.4	42.5 ± 6.9	<0.001	N/A	N/A	N/A
Total dairy intake, g/day	413 ± 320	403 ± 304	0.562	413 ± 445	352 ± 315	0.002	345 ± 211	412 ± 227	<0.001	136 ± 204	195 ± 241	<0.001
			(0.754)			(0.006)			(<0.001)			(<0.001)
Milk intake, g/day	355 ± 301	340 ± 273	0.306	368 ± 427	305 ± 306	0.001	236 ± 182	278 ± 3	<0.001	103 ± 187	155 ± 223	<0.001
			(0.629)			(0.001)			(<0.001)			(<0.001)
Yogurt intake, g/day	22.2 ± 53.2	40.2 ± 69.6	<0.001	15.4 ± 33.0	28.1 ± 40.4	<0.001	66.6 ± 80.2	93.1 ± 92.5	<0.001	27.3 ± 61.6	33.4 ± 66.9	<0.001
			(<0.001)			(<0.001)			(<0.001)			(<0.001)
Cheese intake, g/day	35.3 ± 39.2	23.6 ± 24.7	<0.001	29.8 ± 28.3	18.9 ± 15.8	<0.001	29.1 ± 26.3	30.5 ± 26.6	0.030	4.8 ± 8.0	6.5 ± 11.9	<0.001
			(<0.001)			(<0.001)			(0.010)			(<0.001)
Diabetes,%	39.5	39.5	0.849	6.0	8.7	0.225	53.7	44.4	<0.001	11.9	7.4	<0.001
Current smoker,%	34.1	20.8	<0.001	8.2	8.2	0.911	25.2	5.7	<0.001	4.3	2.0	<0.001
Current drinker,%	49.9	35.6	<0.001	48.8	50.4	0.676	83.6	48.1	<0.001	20.7	18.2	0.003
MCM6-rs3754686 Genotype (%)**												
CC (LNP)	37.2	36.1	0.716	6.7	7.0	0.972	23.6	22.6	0.618	56.3	31.8	<0.001
CT(LP _Heterozygote_ )	45.6	45.7		40.6	41.0		49.1	49.6		36.2	49.5	
TT(LP _Homozygote_ )	17.2	18.2		52.7	52.0		27.3	27.8		7.6	18.7	

*Values are expressed as mean ± standard deviation for continuous variables or as % for categorical variables.

**The rs3754686 SNP was determined in the PREDIMED Study and imputed in the BPRHS. The proxy SNP rs309180 was used in GOLDN and WHI studies.

^1^P-values for differences in sex and differences in race. Chi-squared tests were used to test differences in percentages. We used t-test to compare means of continuous variables. The P-values without parentheses refer to the untransformed continuous variables (except for log-transformed Triglycerides), whereas values in parentheses refer to square-root transformed variables for dairy products.

LNP Lactase Non-persistence; LP_Heterozygote_: Lactase Persistence as heterozygote genotype; LP_Homozygote_: Lactase Persistence as homozygote genotype.

***Some variables (biomarkers and dietary intake) included missing data points.

**Table 2:** Associations of *MCM6-*rs3754686 with dairy and nutrient intake*.

	BPRHS			P	GOLDN			P
	Whole population				Whole population			
	CC (490)	CT (558)	TT (196)		CC (56)	CT (333)	TT (428)	
Total dairy,	372 ± 14	426 ± 13	407 ± 21	0.030	246 ± 28	358 ± 20	377 ± 16	4.2 × 10^−3^
g/day				(0.031)		0.0028	2.80E-03	(2.8 × 10^−3^)
Milk intake,	315 ± 12	366 ± 12	342 ± 20	0.025	199 ± 26	316 ± 19	331 ± 15	2.2 × 10^−3^
g/day				(0.037)		0.0011	1.10E-03	(1.1 × 10^−3^)
Yogurt intake,	32.8 ± 3.0	35.0 ± 2.8	36.3 ± 4.6	0.747	23.2 ± 5.8	18.7 ± 1.6	22.6 ± 1.9	0.494
g/day				(0.663)				(0.515)
Cheese intake,	23.8 ± 1.2	24.6 ± 1.1	28.4 ± 1.8	0.071	24.9 ± 2.8	22.7 ± 1.1	22.5 ± 1.0	0.268
g/day				(0.142)				(0.165)
Calcium, mg/day	1043 ± 27	1092 ± 25	1078 ± 42	0.337	828 ± 57	934 ± 29	942 ± 27	0.218
Total energy intake, kcal/day	2099 ± 40	2112 ± 38	2191 ± 63	0.177	2073 ± 106	2077 ± 46	2003 ± 49	0.210
Total fat, %energy	31.3 ± 0.2	31.1 ± 0.2	31.1 ± 0.4	0.980	37.1 ± 1.1	35.3 ± 0.4	35.5 ± 0.4	0.225
Saturated fat, %energy	9.3 ± 0.1	9.5 ± 0.1	9.6 ± 0.2	0.210	11.9 ± 0.4	11.8 ± 0.2	11.8 ± 0.1	0.866
	PREDIMED				WHI			
	Whole population				Whole population			
	CC (1642)	CT (3518)	TT (1967)	P	CC (5282)	CT (4368)	TT (1193)	P
Total dairy,	359 ± 5	386 ± 3	399 ± 5	1.3 × 10^−6^	127 ± 3	177 ± 3	211 ± 6	1.2 × 10^−46^
g/day				(1.9 × 10^−6^)		3.4E-66	3.4E-66	(3.4 × 10^−66^)
Milk intake,	241 ± 4	261 ± 3	272 ± 4	9.8 × 10^−6^	94.5 ± 2.8	141.2 ± 3.1	169.3 ± 5.8	2.3 × 10^−44^
g/day				(4.0 × 10^−6^)		1.22E-65	1.2E-65	(1.2 × 10^−65^)
Yogurt intake,	77.0 ± 2.1	82.6 ± 1.5	84.5 ± 2.1	0.131	27.5 ± 0.9	30.3 ± 1.0	36.1 ± 1.8	0.078
g/day				(0.171)		0.001859	1.9E-03	(1.9 × 10^−3^)
Cheese intake,	29.7 ± 0.6	29.9 ± 0.5	30.1 ± 0.6	0.206	5.0 ± 0.1	5.7 ± 0.1	5.7 ± 0.3	3.8 × 10^−3^
g/day				(0.266)		2.86E-08	2.9E-08	(2.9 × 10^−8^)
Calcium, mg/day	1019 ± 8	1046 ± 6	1065 ± 8	3.3 × 10^−5^	N/A			
Total energy intake, kcal/day	2253 ± 14	2276 ± 10	2296 ± 14	0.009	1611 ± 11	1651 ± 12	1662 ± 23	0.057
Total fat, %energy	39.5 ± 0.2	39.2 ± 0.1	38.8 ± 0.2	0.298	34.7 ± 0.1	34.6 ± 0.1	33.9 ± 0.2	0.158
Saturated fat, %energy	10.0 ± 0.1	10.0 ± 0.0	9.9 ± 0.1	0.661	10.9 ± 0.0	11.1 ± 0.0	11.0 ± 0.1	0.010

*Values are means ± Standard Error of Mean. P-values adjusted for sex, age, field center or ancestry (BPRHS, WHI), family (GOLDN), BMI, smoking, drinking, physical activity, diabetes, medication and total energy intake. The rs3754686 SNP was determined in the PREDIMED Study and imputed in the BPRHS. The proxy SNP rs309180 was used in GOLDN and WHI studies.

**General Linear Regression models with multivariable adjustment for the indicated covariates were fitted for each population.

***Variables for dairy were used untransformed as well as square-root transformed to improve normality. The P-values without parentheses refer to the untransformed continuous variables, whereas values in parentheses refer to square-root transformed variables for dairy products.

**Table 3:** Associations of *MCM6-*rs3754686 proxy for milk intake with fasting glucose and lipids*.

	BPRHS					GOLDN				
	CC (485)	CT (553)	TT (194)	P^1^	P^2^	CC (56)	CT (333)	TT (428)	P^1^	P^2^
Glucose, mg/dL	127 ± 3	120 ± 2	115 ± 4	0.036	0.032	103 ± 2	103 ± 1	101 ± 1	0.479	0.091
Total cholesterol, mg/dL	181 ± 2	181 ± 2	179 ± 3	0.914	0.968	197 ± 5	195 ± 2	191 ± 2	0.225	0.216
LDL-C, mg/dL	105 ± 2	105 ± 2	105 ± 3	0.981	0.939	127 ± 4	125 ± 2	123 ± 2	0.518	0.470
HDL-C, mg/dL	43.8 ± 0.6	44.3 ± 0.5	41.7 ± 0.9	0.038	0.041	46.9 ± 1.7	46.0 ± 0.7	45.9 ± 0.7	0.855	0.827
Triglycerides, mg/dL	168 ± 6	163 ± 5	169 ± 9	0.637	0.859	144 ± 11	142 ± 6	142 ± 5	0.894	0.827
	PREDIMED					WHI				
	CC (1594)	CT (3399)	TT (1927)	P^1^	P^2^	CC (600)	CT (518)	TT (124)	P^1^	P^2^
Glucose, mg/dL	121 ± 40	123 ± 42	120 ± 41	0.421	0.793	106 ± 1	106 ± 2	103 ± 3	0.562	0.923
Total cholesterol, mg/dL	211 ± 38	211 ± 38	212 ± 42	0.763	0.722	222 ± 2	218 ± 2	220 ± 4	0.277	0.159
LDL-C, mg/dL	131 ± 34	130 ± 34	131 ± 38	0.922	0.551	138 ± 2	134 ± 2	135 ± 3	0.219	0.153
HDL-C, mg/dL	53.3 ± 13.5	53.9 ± 14.0	54.2 ± 14.7	0.045	0.044	57.0 ± 0.6	57.2 ± 0.6	58.0 ± 1.3	0.765	0.756
Triglycerides, mg/dL	139 ± 85	136 ± 75	138 ± 82	0.989	0.591	136 ± 3	135 ± 3	130 ± 6	0.828	0.429

*Values are means ± Standard Error of Mean. The rs3754686 SNP was determined in the PREDIMED Study and imputed in the BPRHS. The proxy SNP rs309180 was used in GOLDN and WHI studies.

**General Linear Regression models with multivariable adjustment for the indicated covariates were fitted for each population.

^1^P adjusted by sex, age, field center or race.

^2^P adjusted for sex, age, field center or ancestry (BPRHS, WHI), family (GOLDN), BMI, smoking, drinking, physical activity, diabetes, medication and total energy intake.

In PREDIMED, some variables (glucose, LDL-C, HDL-C and triglycerides) included missing data point. Biochemical data were available for fasting glucose (n = 6801 participants) total cholesterol (n = 6920 participants), HDL cholesterol (n = 6837 participants), LDL cholesterol (n = 6782 participants), and triglycerides (n = 6881 participants).

**Table 4:** Incidence and hazard ratios (HR) for cardiovascular diseases (CVD) and total mortality depending on the MCM6-rs3754686 proxy for milk intake after 4.8 years of median follow-up in the PREDIMED trial.

	CVD incidence (men + women): n = 7,185										Model 3	Model 4
	Cases	Non-cases	person-y	Incidence rate*	Model 1			Model 2				
					HR	95% CI	P-value	HR	95% CI	P-value	P-value	P-value
MCM6 genotypes**												
TT	74	1908	8612	8.6	1.00	(reference)		1.00	(reference)			
CT	136	3410	15361	8.9	1.04	(0.79–1.39)	0.768	1.02	(0.77–1.36)	0.890	0.941	0.945
CC	57	1600	7016	8.1	0.94	(0.66–1.33)	0.733	0.95	(0.69–1.35)	0.764	0.981	0.815
TT (ref.)***					1.00	(reference)		1.00	(reference)			
(CC + TC) vs TT					1.01	(0.77–1.33)	0.932	1.00	(0.76–1.31)	0.989	0.928	0.969
Per variant allele (T)****					1.03	(0.87–1.22)	0.767	1.02	(0.86–1.22)	0.785	0.792	0.829
*P*^**§**^-interaction sex*MCM6 polymorphism: 0.005												
	**Total mortality (men + women): n = 7,185**											
					Model 1			Model 2			Model 3	Model 4
MCM6 genotypes**												
TT	104	1878	8622	12.1	1.00	(reference)		1.00	(reference)			
CT	139	3407	15375	9.0	0.73	(0.58–0.97)	0.029	0.75	(0.58–0.97)	0.028	0.028	0.030
CC	79	1579	7027	11.2	0.89	(0.86–1.19)	0.424	0.89	(0.66–1.21)	0.464	0.455	0.549
TT (ref.)***					1.00	(reference)		1.00	(reference)			
(CC + CT) vs TT					0.80	(0.63–1.01)	0.057	0.79	(0.62–1.01)	0.058	0.057	0.068
Per variant allele (T)****					1.08	(0.92–1.26)	0.338	1.07	(0.92–1.26)	0.378	0.371	0.446
*P*^**§§**^-interaction sex*MCM6 polymorphism: 0.032												

*Crude incidence rates were expressed per 1000 person-years of follow-up.

**Codominant model. ***Recessive model.****Additive model.

We used multivariable Cox regression models with length of follow-up as the primary time variable. Separate models were fitted for CVD and total mortality to estimate the corresponding HRs depending on the model.

Model 1: Adjusted for sex, age, field center and dietary intervention group.

Model 2: Model 1 adjusted for variables in model 1 plus BMI, diabetes, drinking, smoking, physical activity, medication (hypertension, dyslipidemia and glucose) and total energy intake at baseline.

Model 3: Model 2 adjusted for variables in model 2 plus total milk intake. Model 4: Model 3 additionally adjusted for total fat and carbohydrates at baseline.

^§^*P*-value for interaction sex*MCM6 polymorphism in determining CVD incidence, obtained in Model 2. Further adjustments did not change the statistical significance.

^§§^*P*-value for interaction sex*MCM6 polymorphism in determining mortality, obtained in Model 2. Further adjustments did not change the statistical significance.
