# Supplementary material for: Associations of the MCM6-rs3754686 proxy for milk intake in Mediterranean and American populations with cardiovascular biomarkers, disease and mortality: Mendelian randomization
Source: Sci Rep. 2016 Sep 14;6:33188. doi: 10.1038/srep33188 (PMC5021998; doi:10.1038/srep33188)
Supplement: Supplementary Information [file srep33188-s1.pdf]

Associations of the MCM6-rs3754686 proxy for milk intake in Mediterranean and American populations with cardiovascular biomarkers, disease and mortality: Mendelian randomization

Caren E. Smith, Oscar Coltell, Jose V. Sorlí, Ramón Estruch Miguel Ángel Martínez-González, Jordi Salas-Salvadó, Montserrat Fitó, Fernando Arós,Hassan S. Dashti, Chao Q Lai, Leticia Miró, Lluís Serra-Majem, Enrique Gómez-Gracia, Miquel Fiol, Emilio Ros, Stella Aslibekyan,Bertha Hidalgo, Marian L. Neuhouser, Chongzhi Di, Katherine L. Tucker, Donna K Arnett, Jose M Ordovas, Dolores Corella.

Supplementary Information

Supplementary figures

- Supplementary figure S1. SNP location and linkage disequilibrium (LD) parameters.
- Supplementary figure S2. Meta-analysis of the association between the MCM6-rs3754686 polymorphism and total dairy intake (A), total milk (B), yogurt (C) and cheese (D) in participants (men and women) of the BPRHS, GOLDN, PREDIMED and WHI studies (n=20031).
- Supplementary figure S3. Meta-analysis of the association between the MCM6-rs3754686 polymorphism and total dairy intake according to sex in BPRHS, GOLDN, PREDIMED and WHI studies.
- Supplementary figure S4. Longitudinal effect of the *MCM6*-rs3754686 polymorphism on total dairy intake over a 5-y follow-up period in the PREDIMED study in men and women combined.
- Supplementary figure S5. Meta-analysis of the association between total milk intake and fasting glucose according to sex in BPRHS, GOLDN, PREDIMED and WHI studies.
- Supplementary figure S6. Kaplan Meier curves of cumulative CVD-free survival or mortality-free survival in women depending on the MCM6-rs3754686 polymorphism and dietary intervention group (Mediterranean diet vs control diet) in the PREDIMED participants.
- Supplementary figure S7. Kaplan Meier curves of cumulative CVD-free survival or mortality-free survival in women depending on total milk intake in the PREDIMED participants.

Supplementary tables

- Supplementary table S1. Associations of MCM6-rs3754686 with population characteristics (potential confounding factors)*.
- Supplementary table S2. Descriptives of milk type intake by sex in BPRHS, GOLDN, PREDIMED and WHI studies.
- Supplementary table S3. Associations of MCM6-rs3754686 with dietary intake in women in the studied populations.
- Supplementary table S4. Associations of MCM6-rs3754686 with dietary intake in men in the studied populations.
- Supplementary table S5. Associations of MCM6-rs3754686 proxy for milk intake with fasting glucose and lipids in men in the studied populations.

- Supplementary table S6. Associations of MCM6-rs3754686 proxy for milk intake with fasting glucose and lipids in women in the studied populations.
- Supplementary table S7. Associations of milk intake with fasting glucose and lipids in the whole population and stratified by sex or race in BPRHS, GOLDN, PREDIMED and WHI studies.
- Supplementary table S8. Incidence and hazard ratios (HR) for CVD depending on the MCM6-rs3754686 polymorphism after 4.8 years of median follow-up and stratified by sex.
- Supplementary table S9. Incidence and hazard ratios (HR) for total mortality depending on the MCM6-rs3754686 polymorphism after 4.8 years of median follow-up and stratified by sex.
- Supplementary table S10. Incidence and hazard ratios (HR) for CVD depending on the MCM6-rs3754686 polymorphism after 4.8 years of median follow-up for the Mediterranean Diet intervention group and stratified by sex.
- Supplementary table S11. Incidence and hazard ratios (HR) for CVD depending on the MCM6-rs3754686 polymorphism after 4.8 years of median follow-up for the Control group and stratified by sex.
- Supplementary table S12. Incidence and hazard ratios (HR) for total mortality depending on the MCM6-rs3754686 polymorphism after 4.8 years of median follow-up for the Mediterranean Diet intervention group and stratified by sex.
- Supplementary table S13. Incidence and hazard ratios (HR) for total mortality depending on the MCM6-rs3754686 polymorphism after 4.8 years of median follow-up for the Control group and stratified by sex.
- Supplementary table S14. Incidence and hazard ratios (HR) for CVD incidence depending on milk intake and stratified by sex.
- Supplementary table S15. Description, design and protocols of the BPRHS, GOLDN, PREDIMED and WHI studies.

Supplementary figure S1: SNP location and linkage disequilibrium (LD) parameters. Physical map showing the location of the analyzed *MCM6*-rs3754686 SNP and the LD parameters: (A) Calculated R^2 for the *MCM6*-rs3754686 SNP with another ten *MCM6*-SNPs (including the classic SNP rs4988235) and four LCT-SNPs in a subsample of the PREDIMED study. Genotyping was carried out by using the HumanOmniExpress BeadChip (Illumina) in a subsample consisting of 1020 randomly selected PREDIMED-Valencia (one of the PREDIMED field centers) participants. LD was assessed with the Haploview software package (version 4.2; Broad Institute). In this array, the *MCM6*-rs309180 (a proxy used in GOLDN and WHI studies) was not included and the LD parameters were not available in (A); (B) SNP location and distances for the MCM6-rs3754686, its proxy *MCM6*-rs309180, and the classic rs4988235, obtained from the NCBI Gene database and the corresponding Variation Viewer (GRCh38). In addition, LD parameters among these SNPs, obtained in different populations from Hapmap 3 (release 2), have been included.

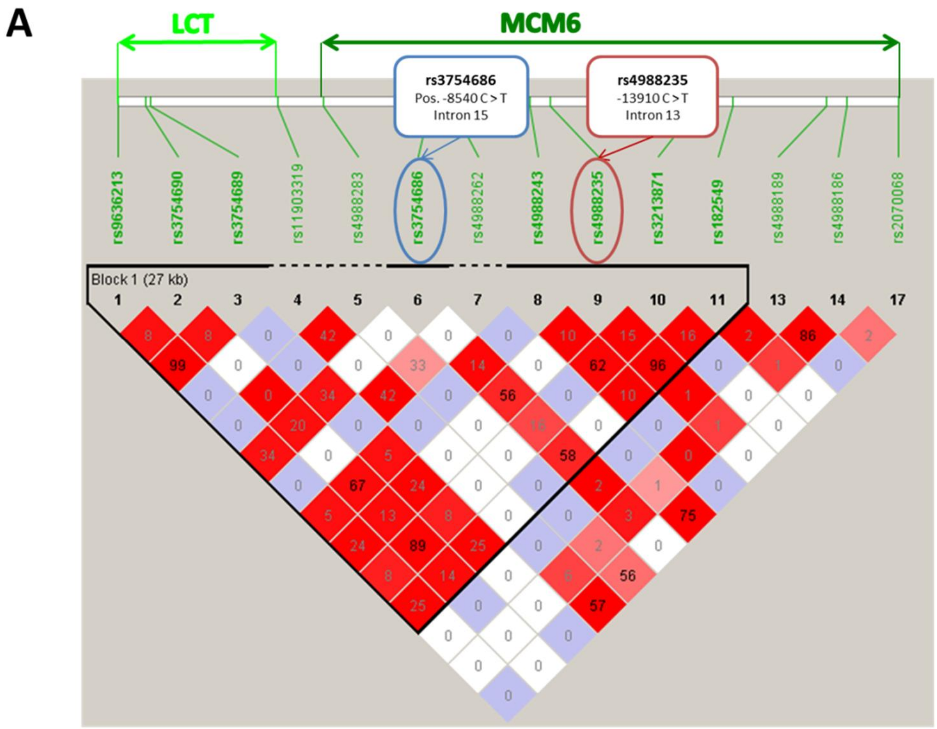

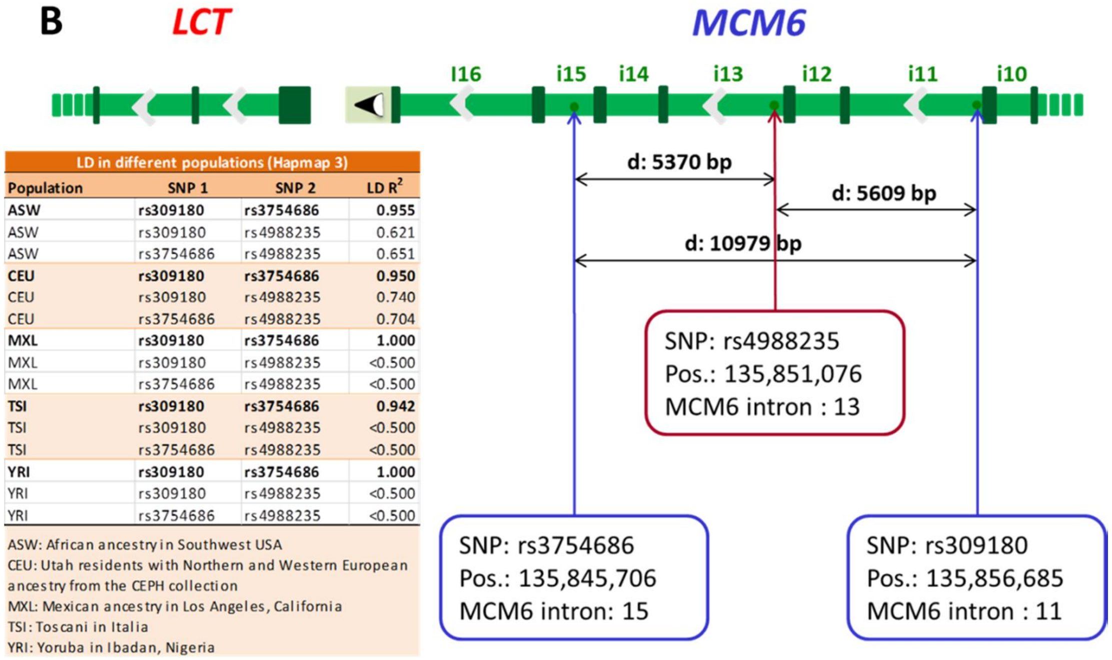

LD in different populations (Hapmap 3)			
Population	SNP 1	SNP 2	LD R^2
**ASW**	**rs309180**	**rs3754686**	**0.955**
ASW	rs309180	rs4988235	0.621
ASW	rs3754686	rs4988235	0.651
**CEU**	**rs309180**	**rs3754686**	**0.950**
CEU	rs309180	rs4988235	0.740
CEU	rs3754686	rs4988235	0.704
**MXL**	**rs309180**	**rs3754686**	**1.000**
MXL	rs309180	rs4988235	<0.500
MXL	rs3754686	rs4988235	<0.500
**TSI**	**rs309180**	**rs3754686**	**0.942**
TSI	rs309180	rs4988235	<0.500
TSI	rs3754686	rs4988235	<0.500
**YRI**	**rs309180**	**rs3754686**	**1.000**
YRI	rs309180	rs4988235	<0.500
YRI	rs3754686	rs4988235	<0.500

ASW: African ancestry in Southwest USA
CEU: Utah residents with Northern and Western European ancestry from the CEPH collection
MXL: Mexican ancestry in Los Angeles, California
TSI: Toscani in Italia
YRI: Yoruba in Ibadan, Nigeria

Supplementary figure S2: Meta-analysis of the association between the MCM6-rs3754686 polymorphism and total dairy intake (A), total milk (B), yogurt (C) and cheese (D) in participants (men and women) of the BPRHS, GOLDN, PREDIMED and WHI studies (n=20,031). Forest plots show adjusted regression coefficients and 95% CI (expressed in g/d and estimated per one copy of the T-allele; LCT genotypes coded as 0, 1, and 2 according to the number of T-alleles) for the corresponding intake in each study. The rs3754686 SNP was determined in PREDIMED and imputed in BPRHS. The proxy rs309180 was genotyped in GOLDN and WHI studies. The diamond shows the meta-analysis associations (weighted average) in a random effects model. The I^2 statistic was calculated for heterogeneity. $P_{meta\text{-}analysis}$ indicates the P-value obtained in the meta-analysis including all populations. $P'_{meta\text{-}analysis}$ indicates the P-value for the meta-analysis obtained in the sensitivity analysis excluding the WHI AA women. In both cases, results for raw data and (square root transformed data) are presented.

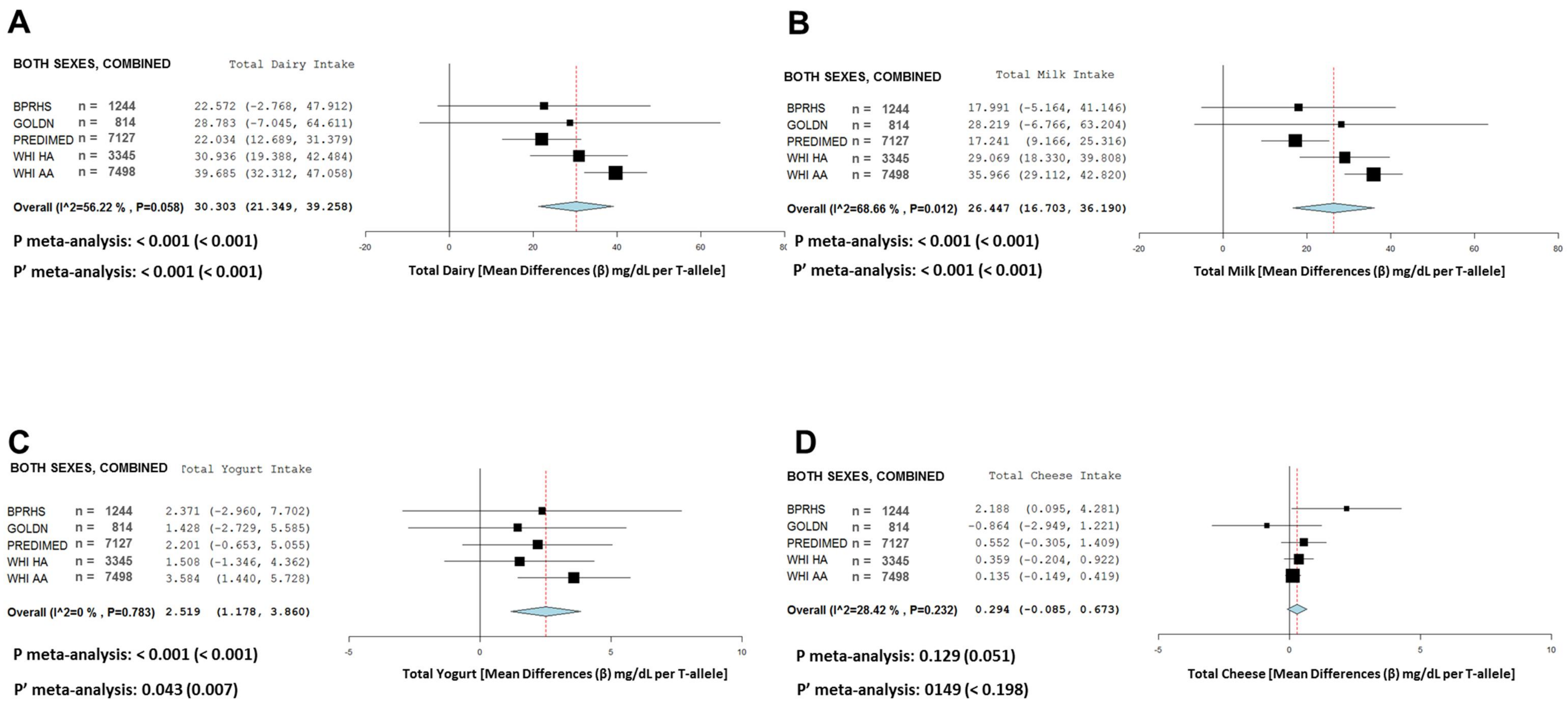

Supplementary figure S3: Meta-analysis of the association between the MCM6-rs3754686 polymorphism and total dairy intake according to sex in BPRHS, GOLDN, PREDIMED and WHI studies. Forest plots: (A) total dairy in men, and (B) total dairy in women, show adjusted regression coefficients and 95% CI (expressed in g/d and estimated per one copy of the T-allele; LCT genotypes coded as 0, 1, and 2 according to the number of T-alleles) for the corresponding intake in each study. The rs3754686 SNP was determined in PREDIMED and imputed in BPRHS. The proxy rs309180 was genotyped in GOLDN and WHI studies. The diamond shows the meta-analyzed associations in a fixed-effects model. The I^2 statistic was calculated for heterogeneity. $P_{meta\text{-}analysis}$ indicates the P-value obtained in the meta-analysis including all populations. $P'_{meta\text{-}analysis}$ indicates the P-value for the meta-analysis obtained in the sensitivity analysis excluding the WHI AA women. In both cases, results for raw data and square root transformed data (values in parentheses) for dairy are presented. P and P' for sex differences indicate the P-values for heterogeneity by sex in the total (P) and the sensitivity (P') meta-analysis.

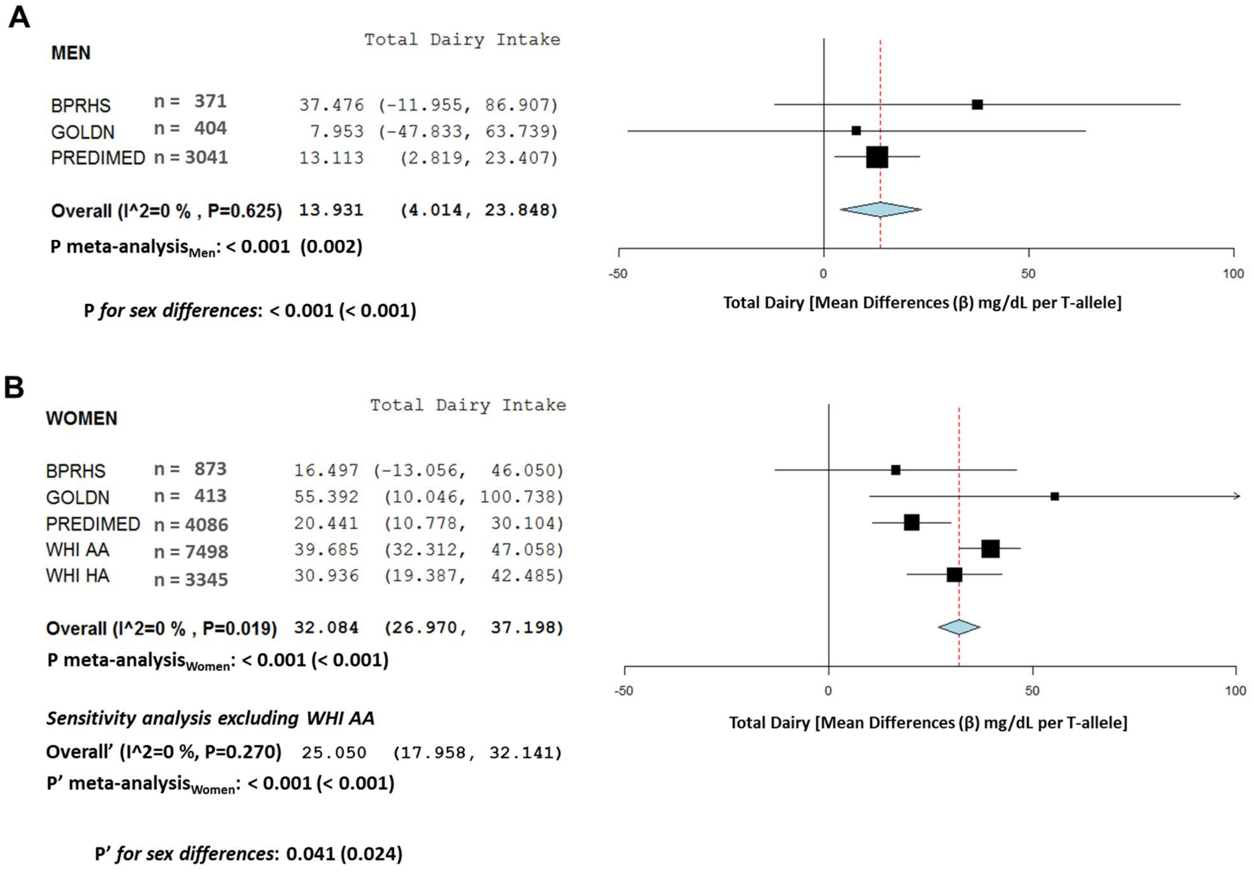

Supplementary figure S4. Longitudinal effect of the *MCM6*-rs3754686 polymorphism on total dairy intake over a 5-y follow-up period in the PREDIMED study in men and women combined. Adjusted means of dairy intake are expressed in g/d yearly depending on the genotype in all subjects having data for all the measurements (n=2,087). Error bars indicate the standard error of means. P-values for the overall effect of the polymorphism as well as the P-values for the interaction term between the MCM6 SNP and sex, were estimated from a repeated-measures ANOVA model adjusted for sex, age, field center, diabetes, smoking, drinking, and total energy intake. The P-values without parentheses refer to the untransformed continuous variables, whereas values in parentheses refer to square root transformed variables for dairy.

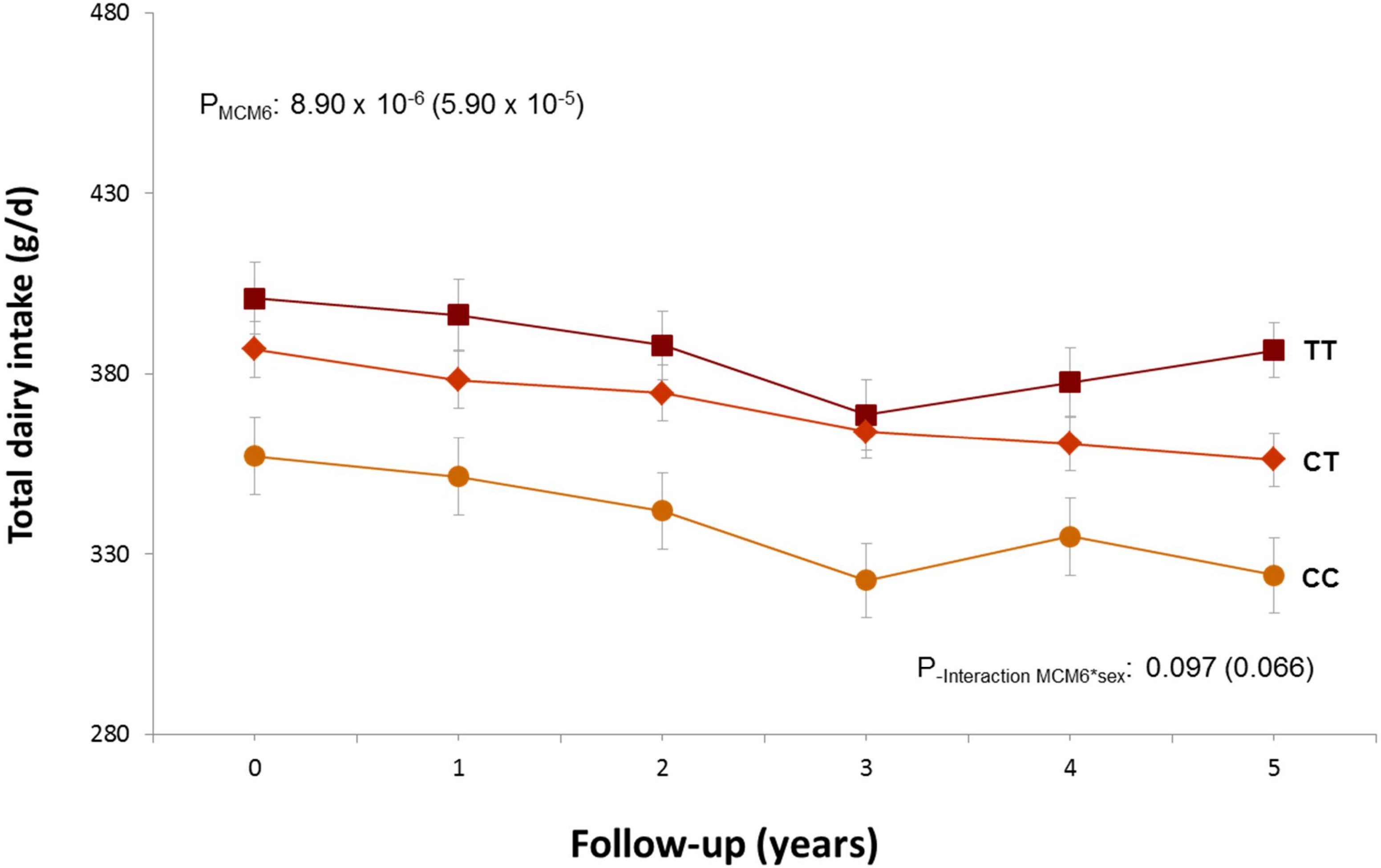

Supplementary figure S5: Meta-analysis of the association between total milk intake and fasting glucose according to sex in BPRHS, GOLDN, PREDIMED and WHI studies (n=10,223). Forest plot in men (M) and women (W), shows adjusted regression coefficients and 95% CI (expressed in mg/dL and estimated per 100 g/d milk intake) for the corresponding intake in each study. The diamond shows the meta-analyzed associations in a fixed-effects model. The I^2 statistic was calculated for heterogeneity. $P_{meta\text{-}analysis}$ indicates the P-value obtained in the meta-analysis including all populations. $P'_{meta\text{-}analysis}$ indicates the P-value for the meta-analysis obtained in the sensitivity analysis excluding the WHI AA women. P and P' for sex differences indicate the P-values for heterogeneity by sex in the total (P) and the sensitivity (P') meta-analysis. In both cases, results for raw data and square root transformed data (values in parentheses) for milk are presented.

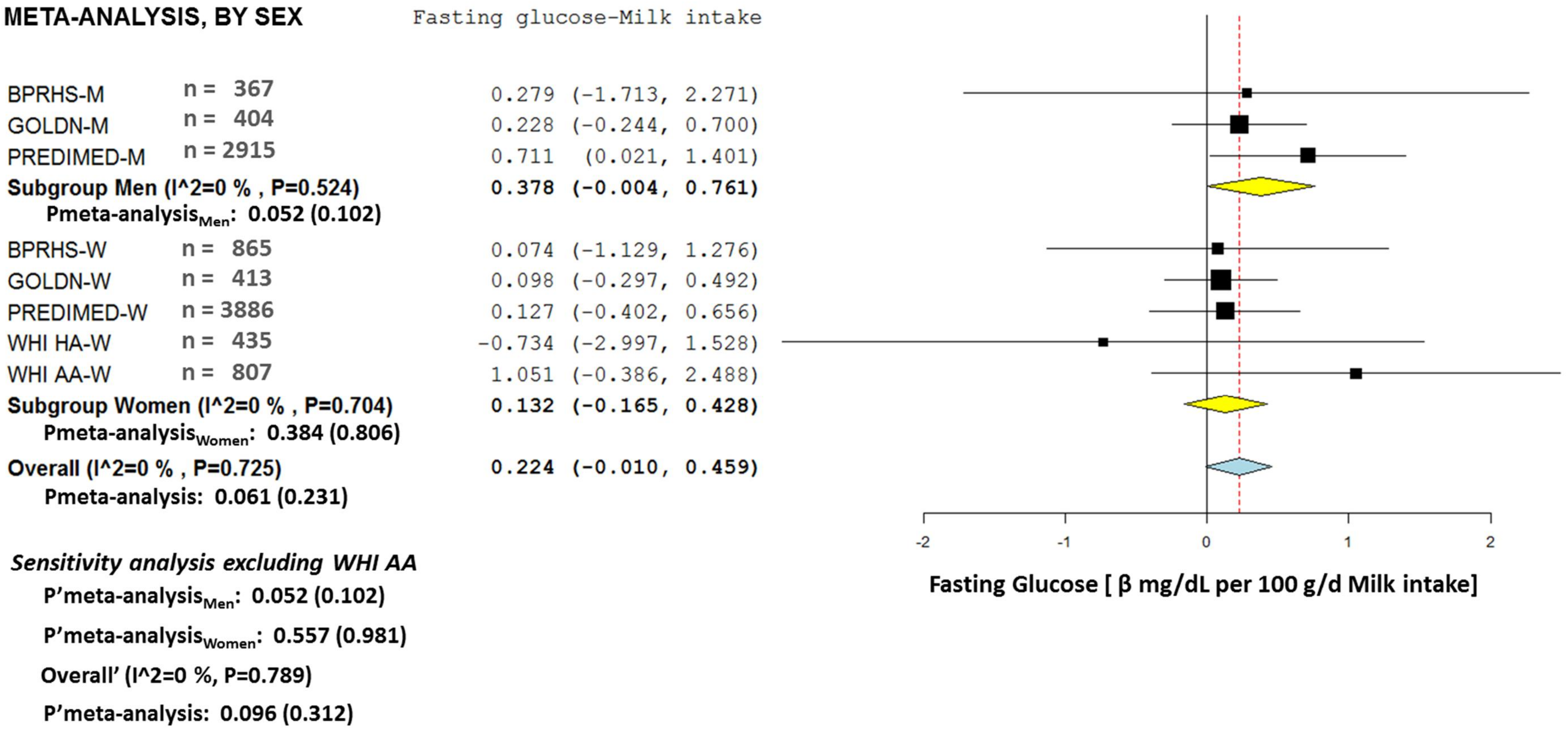

Supplementary figure S6: Kaplan Meier curves of cumulative CVD-free survival or mortality-free survival in women depending on the MCM6-rs3754686 polymorphism and dietary intervention group (Mediterranean diet vs control diet) in the PREDIMED participants. N=4,120 women were analyzed: (A) CVD incidence in the Mediterranean diet group, (B) CVD incidence in the control group,(C) total mortality in the Mediterranean diet group and (D), total mortality in the control group. Multivariable Cox regression models with outcome of CVD incidence or total mortality were fitted as indicated in methods. HR and 95%CI were obtained in the multivariable adjusted models: HR^1: Model 1 (adjusted for sex, age, field center and dietary intervention group) and HR^2: Model 2 (adjusted for variables in model 1 plus BMI, diabetes, drinking, smoking, physical activity, medication (hypertension, dyslipemia and glucose) and total energy intake at baseline).

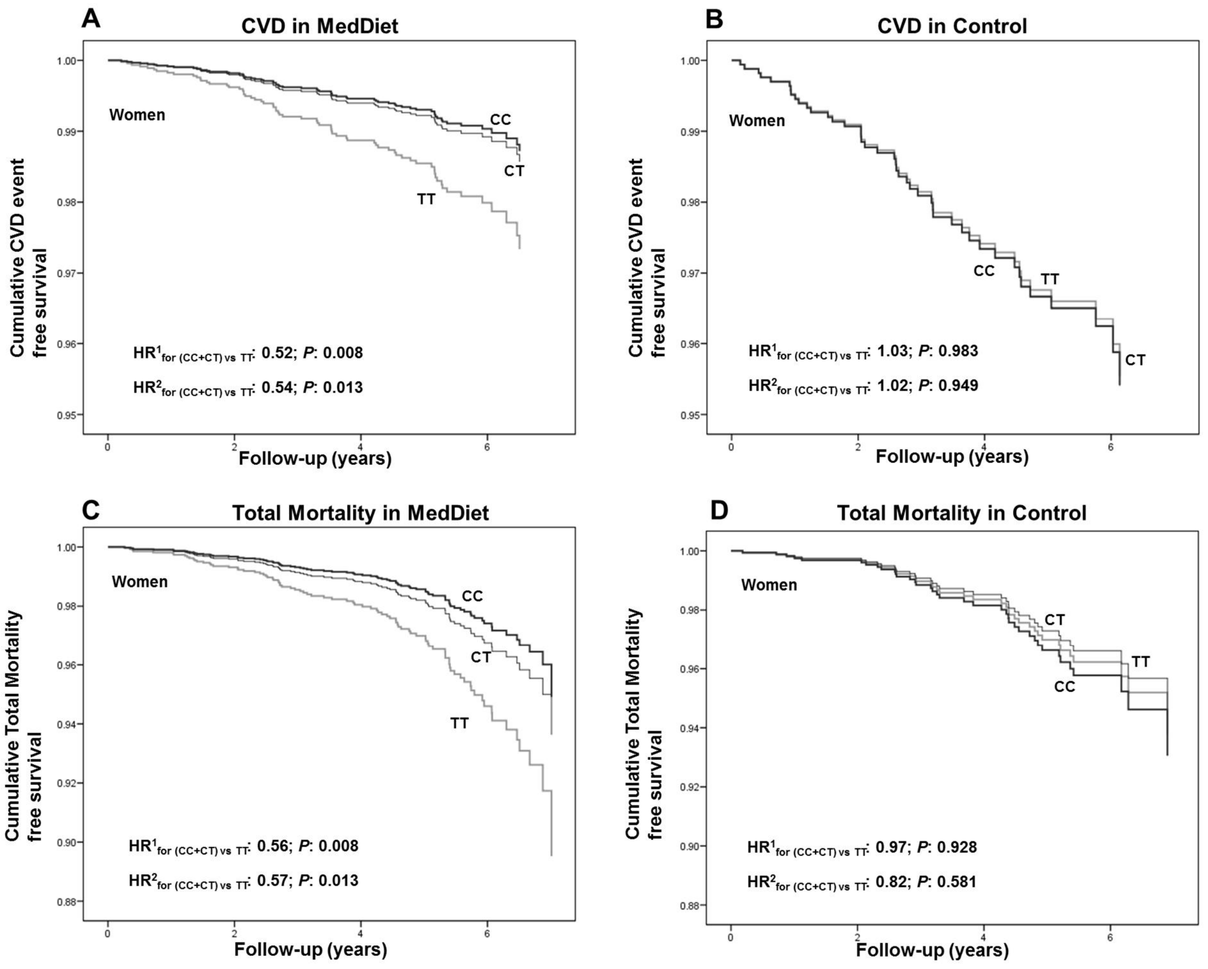

Supplementary figure S7: Kaplan Meier curves of cumulative CVD-free survival or mortality-free survival in women depending on total milk intake in the PREDIMED participants. N=4,089 women were analyzed: (A) CVD incidence in the Mediterranean diet group, (B) CVD incidence in the control group,(C) total mortality in the Mediterranean diet group and (D), total mortality in the control group. Milk was considered as dichotomous based on the population median: 200 g/d). Multivariable Cox regression models with outcome of CVD incidence or total mortality were fitted as indicated in methods. HR and 95%CI were obtained in the multivariable adjusted models: HR^a: Model 1 (adjusted for sex, age, field center and dietary intervention group) and HR^b: Model 2 (adjusted for variables in model 1 plus BMI, diabetes, drinking, smoking, physical activity, medication (hypertension, dyslipemia and glucose) and total energy intake at baseline).

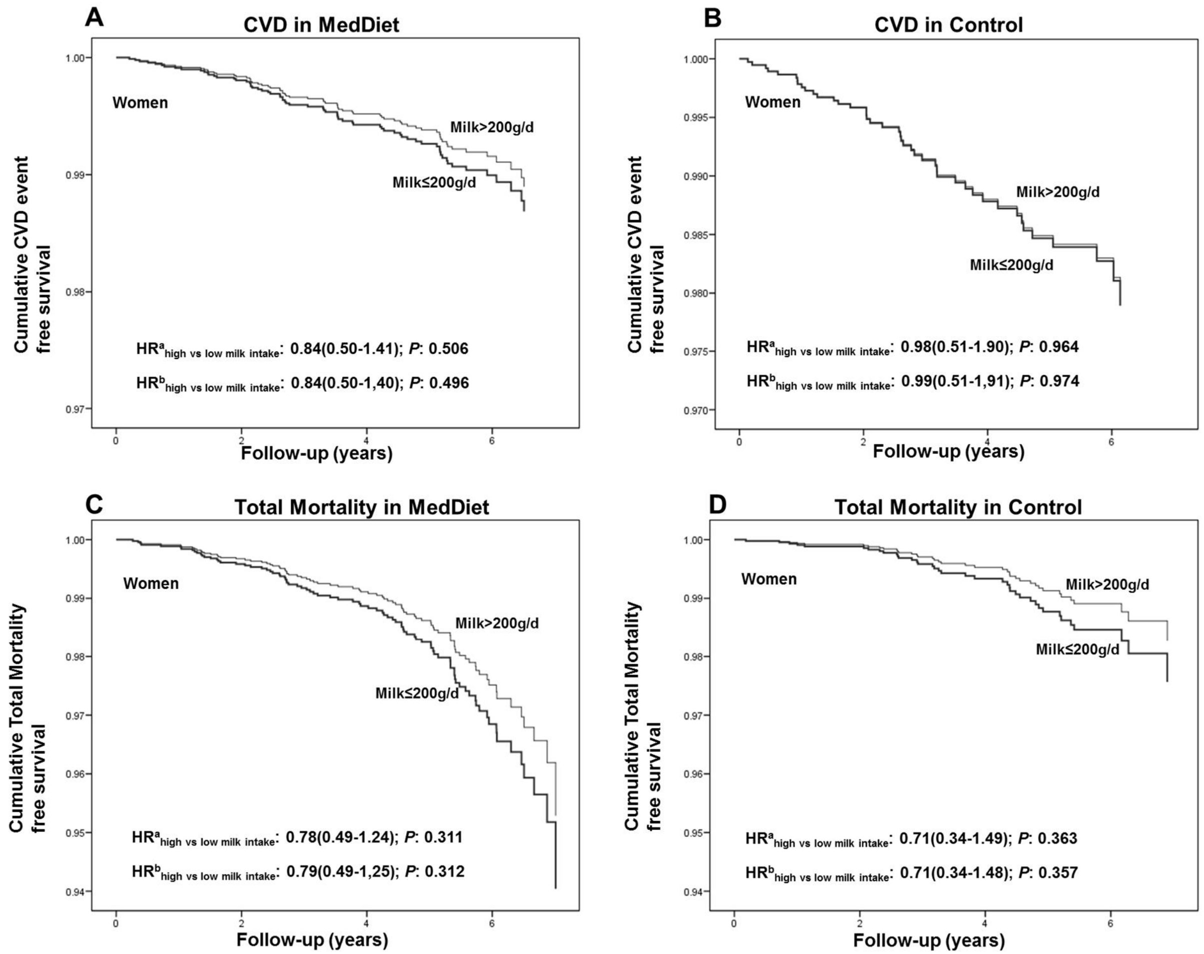

Supplemental table S1. Associations of MCM6-rs3754686 with population characteristics (potential confounding factors)*

	BPRHS				GOLDN				PREDIMED			
	CC (490)	CT (558)	TT (196)	P[1]	CC (29)	CT (169)	TT (215)	P[1]	CC (1657)	CT (3546)	TT (1982)	P[1]
Age, yrs	56.9 ± 0.4	57.5 ± 0.3	56.8 ± 0.6	0.291	49.0 ± 2.1	49.5 ± 0.9	48.4 ± 0.8	0.649	67.1 ± 0.2	67.0 ± 0.1	66.9 ± 0.1	0.653
BMI, kg/m^2	31.6 ± 0.3	32.0 ± 0.3	31.8 ± 0.5	0.719	28.3 ± 0.7	28.3 ± 0.3	28.6 ± 0.3	0.791	29.9 ± 0.1	29.9 ± 0.1	30.1 ± 0.1	0.166
Height , m	1.59 ± 0.00	1.58 ± 0.00	1.58 ± 0.01	0.299	1.72 ± 0.01	1.71 ± 0.01	1.71 ± 0.00	0.988	1.60 ± 0.00	1.60 ± 0.00	1.60 ± 0.00	0.509
Sex, % males	31	29	31	0.716	52	51	50	0.972	44	42	42	0.618
Current smoker,%	25	24	27	0.745	7	8	9	0.877	14	14	15	0.386
Current drinker,%	42	39	37	0.527	55	52	47	0.318	65	63	64	0.447
Diabetes, %	41	38	41	0.480	9	7	8	0.799	48	50	45	0.001

	WHI							
	African American				Hispanic American			
	CC (4219)	CT (2712)	TT (567)	P[1]	CC (1063)	CT (1656)	TT (626)	P[1]
Age, yrs	60.9 ± 0.1	61.3 ± 0.1	61.9 ± 0.3	< 0.001	60.2 ± 0.2	59.7 ± 0.2	60.4 ± 0.3	0.063
BMI, kg/m^2	31.1 ± 0.1	30.7 ± 0.1	30.6 ± 0.3	0.020	28.6 ± 0.2	28.9 ± 0.1	28.8 ± 0.2	0.302
Height , m	1.63 ± 0.00	1.62 ± 0.00	1.63 ± 0.00	0.224	1.57 ± 0.00	1.58 ± 0.00	1.58 ± 0.00	0.359
Sex, % males	0	0	0		0	0	0	
Current smoker,%	4	5	4	0.529	2	2	2	0.977
Current drinker,%	21	20	18	0.155	17	18	19	0.483
Diabetes, %	12	11	12	0.264	9	7	5	0.007

*: Values are expressed as mean ± standard deviation for continuous variables or as % for categorical variables. The rs3754686 SNP was determined in PREDIMED and imputed in BPRHS. The proxy rs309180 was genotyped in GOLDN and WHI studies.

[1]: P-values for differences in sex and differences in race. Chi-squared tests were used to test differences in percentages. We used ANOVA test to compare means of continuous variables.

Supplemental table S2. Descriptives of milk type intake by sex in BPRHS, GOLDN, PREDIMED and WHI studies

	BPRHS	GOLDN	PREDIMED	WHI African-American	WHI Hispanic-American
	Mean (SD)	Mean (SD)	Mean (SD)	Mean (SD)	Mean (SD)
Whole milk (g/d)					
Men	148.8 (174.1)	48.0 (92.0)	56.7 (131.5)		
Women	126.7 (127.4)	32.4 (63.4)	39.6 (117.8)	45.1 (95.0)	43.7 (93.8)
Reduced fat milk (1%, 2%) (g/d)					
Men	111.8 (183.7)	195.1 (189.3)	96.4 (164.6)		
Women	114.4 (148.3)	177.3 (175.8)	120.3 (186.9)	124.4 (133.0)	131.4 (132.7)
Skim milk (non fat) (g/d)					
Men	47.6 (90.4)	103.2 (164.9)	83.3 (153.7)		
Women	53.9 (103.3)	105.8 (153.9)	118.1 (187.4)	70.4 (110.9)	73.8 (112.4)

*: Values are expressed as mean ± standard deviation.

BPRHS (men=371, women=873); GOLDN (men=404, women=413); PREDIMED (men=3041, women=4086); WHI (African-American=7498, Hispanic-American=3345).

Supplemental table S3. Associations of MCM6-rs3754686 with dietary intake in women in the studied populations

	BPRHS					GOLDN					PREDIMED				
	CC (339)	CT (398)	TT (136)	P[1]	P[2]	CC (29)	CT (169)	TT (215)	P[1]	P[2]	CC (922)	CT (2027)	TT (1137)	P[1]	P[2]
Total dairy, g/day	380 ± 17	421 ± 15	401 ± 26	0.190	0.222 (0.200)	209 ± 27	329 ± 25	401 ± 24	0.001	0.003 (0.005)	383 ± 7	414 ± 5	433 ± 7	<0.001	3.4×10^{-5} (6.5×10^{-5})
Milk intake, g/day	319 ± 15	360 ± 14	332 ± 23	0.126	0.154 (0.162)	155 ± 22	286 ± 24	349 ± 23	4.0×10^{-4}	0.001 (0.003)	255 ± 6	280 ± 4	293 ± 6	<0.001	1.9×10^{-4} (2.7×10^{-4})
Yogurt intake, g/day	39 ± 4	38 ± 4	43 ± 6	0.791	0.652 (0.822)	36 ± 12	25 ± 3	31 ± 3	0.225	0.201 (0.259)	87.0 ± 3.0	93.4 ± 2.0	97.6 ± 2.8	0.010	0.091 (0.057)
Cheese intake, g/day	21.3 ± 1.3	22.8 ± 1.2	26.1 ± 2.0	0.140	0.087 0.129	17.5 ± 2.3	18.0 ± 1.1	19.9 ± 1.2	0.360	0.609 (0.788)	31.0 ± 0.8	30.3 ± 0.6	30.6 ± 0.8	0.742	0.635 (0.419)
Calcium, mg/day	1064 ± 33	1097 ± 31	1061 ± 52	0.724	0.685	736 ± 63	827 ± 36	935 ± 35	0.024	0.039	1028 ± 12	1062 ± 8	1089 ± 12	<0.001	2.3×10^{-4}
Total energy intake, kcal/day	1961 ± 48	2014 ± 44	2081 ± 74	0.382	0.138	1792 ± 101	1745 ± 49	1828 ± 54	0.504	0.606	2121 ± 18	2148 ± 12	2178 ± 17	0.022	0.005
Total fat, % energy	31 ± 0	31 ± 0	31 ± 0	0.964	0.977	36 ± 1	35 ± 1	35 ± 1	0.471	0.404	39.9 ± 0.2	39.4 ± 0.2	39.2 ± 0.2	0.014	0.068
Saturated fat, % energy	9 ± 0	9 ± 0	9 ± 0	0.389	0.250	12 ± 0	11 ± 0	12 ± 0	0.673	0.744	10.1 ± 0.1	10.0 ± 0.1	10.0 ± 0.1	0.137	0.895

	WHI									
	African American					Hispanic American				
	CC (4219)	CT (2712)	TT (567)	P[1]	P[2]	CC (1063)	CT (1656)	TT (626)	P[1]	P[2]
Total dairy, g/day	116 ± 3	163 ± 4	187 ± 9	7.5×10^{-27}	8.0×10^{-26} (4.3×10^{-34})	170 ± 7	200 ± 6	233 ± 9	1.7×10^{-7}	1.6×10^{-7} (2.3×10^{-10})
Milk intake, g/day	86 ± 3	129 ± 4	150 ± 8	8.4×10^{-26}	1.2×10^{-24} (3.7×10^{-33})	129 ± 7	162 ± 5	186 ± 9	1.3×10^{-7}	1.2×10^{-7} (4.5×10^{-11})
Yogurt intake, g/day	4.8 ± 0.1	5.0 ± 0.2	4.8 ± 0.3	0.468	0.407 (0.200)	5.9 ± 0.4	6.8 ± 0.3	6.5 ± 0.5	0.130	0.146 (0.116)
Cheese intake, g/day	25.6 ± 0.9	29.7 ± 1.2	31.8 ± 2.5	0.005	0.004 (7.4×10^{-5})	35.3 ± 2.1	31.2 ± 1.7	39.9 ± 2.8	0.022	0.017 (0.233)
Calcium, mg/day	N/A					N/A				
Total energy intake, kcal/day	1598 ± 12	1648 ± 15	1633 ± 33	0.031	0.017	1664 ± 25	1656 ± 20	1688 ± 32	0.683	0.559
Total fat, % energy	35.0 ± 0.1	34.9 ± 0.2	34.4 ± 0.4	0.313	0.414	33.6 ± 0.3	34.0 ± 0.2	33.3 ± 0.3	0.153	0.188
Saturated fat, % energy	11.0 ± 0.0	11.1 ± 0.1	11.1 ± 0.1	0.456	0.366	10.7 ± 0.1	11.1 ± 0.1	11.0 ± 0.1	0.002	0.002

*: Values are means ± Standard Error of Mean. The rs3754686 SNP was determined in PREDIMED and imputed in BPRHS. The proxy rs309180 was genotyped in GOLDN and WHI studies.

[1]: P-values adjusted for sex, age, field center or ancestry (BPRHS, WHI), family (GOLDN), BMI, smoking, drinking, physical activity, diabetes, medication and total energy intake.

[2]: P adjusted for sex, age, field center or ancestry (BPRHS, WHI), family (GOLDN), BMI, smoking, drinking, physical activity, diabetes, medication and total energy intake.

**: General Linear Regression models with multivariable adjustment for the indicated covariates were fitted for each population.

***: Variables for dairy were used untransformed as well as square-root transformed to improve normality. The P-values without parentheses refer to the untransformed continuous variables, whereas values in parentheses refer to square-root transformed variables for dairy products.

Supplemental table S4. Associations of MCM6-rs3754686 with dietary intake in men in the studied populations

	BPRHS					GOLDN					PREDIMED				
	CC (151)	CT (160)	TT (60)	P[1]	P[2]	CC (27)	CT (164)	TT (213)	P[1]	P[2]	CC (720)	CT (1491)	TT (830)	P[1]	P[2]
Total dairy, g/day	356 ± 27	441 ± 27	420 ± 43	0.075	0.100 (0.135)	289 ± 53	394 ± 29	370 ± 23	0.296	0.221 (0.195)	328 ± 7	348 ± 6	355 ± 8	0.015	0.013 (0.009)
Milk intake, g/day	309 ± 25	385 ± 25	365 ± 41	0.097	0.118 (0.195)	246 ± 53	353 ± 29	329 ± 22	0.287	0.225 (0.195)	224 ± 6	237 ± 5	245 ± 6	0.026	0.022 (0.034)
Yogurt intake, g/day	18.3 ± 4.2	26.6 ± 4.1	20.5 ± 6.7	0.358	0.392 (0.195)	11.8 ± 3.0	13.8 ± 2.2	15.6 ± 2.6	0.596	0.502 (0.195)	64.2 ± 2.9	67.8 ± 2.1	66.5 ± 2.9	0.572	0.813 (0.901)
Cheese intake, g/day	29.4 ± 2.4	29.6 ± 2.4	35.0 ± 3.9	0.420	0.632 (0.195)	32.5 ± 4.2	28.1 ± 1.7	25.7 ± 1.5	0.312	0.358 (0.195)	28.2 ± 0.9	29.5 ± 0.7	29.4 ± 0.9	0.365	0.166 (0.150)
Calcium, mg/day	995 ± 46	1086 ± 45	1116 ± 74	0.246	0.415	940 ± 88	1056 ± 41	977 ± 38	0.257	0.166	1009 ± 13	1025 ± 9	1034 ± 13	0.183	0.047
Total energy intake, kcal/day	2424 ± 73	2371 ± 72	2450 ± 116	0.802	0.896	2424 ± 147	2456 ± 64	2253 ± 64	0.039	0.031	2422 ± 23	2451 ± 16	2459 ± 22	0.248	0.378
Total fat, %energy	32.3 ± 0.4	32.0 ± 0.4	32.0 ± 0.7	0.850	0.733	38.1 ± 1.5	36.0 ± 0.6	35.9 ± 0.5	0.436	0.434	38.9 ± 0.3	38.9 ± 0.2	38.3 ± 0.2	0.120	0.298
Saturated fat, %energy	9.6 ± 0.2	9.9 ± 0.2	10.0 ± 0.3	0.580	0.863	12.2 ± 0.5	12.2 ± 0.2	12.1 ± 0.2	0.918	0.916	9.9 ± 0.1	10.0 ± 0.1	9.9 ± 0.1	0.659	0.424

*: Values are means ± Standard Error of Mean. The rs3754686 SNP was determined in PREDIMED and imputed in BPRHS. The proxy rs309180 was genotyped in GOLDN and WHI studies.

[1]: P-values adjusted for sex, age, field center or ancestry (BPRHS, WHI), family (GOLDN), BMI, smoking, drinking, physical activity, diabetes, medication and total energy intake.

[2]: P adjusted for sex, age, field center or ancestry (BPRHS, WHI), family (GOLDN), BMI, smoking, drinking, physical activity, diabetes, medication and total energy intake.

**: General Linear Regression models with multivariable adjustment for the indicated covariates were fitted for each population.

***: Variables for dairy were used untransformed as well as square-root transformed to improve normality. The P-values without parentheses refer to the untransformed continuous variables, whereas values in parentheses refer to square-root transformed variables for dairy products.

Supplemental table S5. Associations of MCM6-rs3754686 proxy for milk intake with fasting glucose and lipids in men in the studied populations

	BPRHS					GOLDN					PREDIMED				
	CC (485)	CT (553)	TT (194)	P[1]	P[2]	CC (56)	CT (333)	TT (428)	P[1]	P[2]	CC (692)	CT (1448)	TT (816)	P[1]	P[2]
Glucose, mg/dL	126 ± 5	120 ± 4	123 ± 7	0.658	0.991	104 ± 2	107 ± 2	105 ± 1	0.401	0.108	125 ± 39	127 ± 42	126 ± 44	0.697	0.140
Total cholesterol, mg/dL	177 ± 4	173 ± 3	164 ± 6	0.124	0.128	199 ± 5	195 ± 3	189 ± 3	0.062	0.025	205 ± 38	202 ± 37	203 ± 43	0.499	0.694
LDL-C, mg/dL	103 ± 3	100 ± 3	94 ± 5	0.288	0.859	129 ± 5	127 ± 2	124 ± 2	0.567	0.360	128 ± 34	126 ± 33	127 ± 41	0.639	0.570
HDL-C, mg/dL	41.1 ± 1.0	40.9 ± 1.0	37.0 ± 1.6	0.074	0.049	43.3 ± 1.9	40.6 ± 0.9	40.7 ± 0.7	0.407	0.368	48.8 ± 11.7	49.3 ± 12.5	48.9 ± 11.8	0.989	0.946
Triglycerides, mg/dL	183 ± 12	169 ± 12	171 ± 19	0.793	0.702	166 ± 18	158 ± 10	151 ± 8	0.545	0.394	144 ± 90	139 ± 82	142 ± 86	0.487	0.259

*: Values are means ± Standard Error of Mean. The rs3754686 SNP was determined in PREDIMED and imputed in BPRHS. The proxy rs309180 was genotyped in GOLDN and WHI studies.

**: General Linear Regression models with multivariable adjustment for the indicated covariates were fitted for each population.

1: P adjusted by sex, age, field center or race.

2: P adjusted for sex, age, field center or ancestry (BPRHS, WHI), family (GOLDN), BMI, smoking, drinking, physical activity, diabetes, medication and total energy intake.

In PREDIMED, some variables (glucose, LDL-C, HDL-C and triglycerides) included missing data point. Biochemical data were available for fasting glucose (n = 2915 participants) total cholesterol (n = 2956 participants), HDL cholesterol (n = 2925 participants), LDL cholesterol (n = 2899 participants), and triglycerides (n = 2946 participants).

Supplemental table S6. Associations of MCM6-rs3754686 proxy for milk intake with fasting glucose and lipids in women in the studied populations

	BPRHS					GOLDN					PREDIMED				
	CC (336)	CT (394)	TT (135)	P1	P2	CC (29)	CT (169)	TT (215)	P1	P2	CC (902)	CT (1951)	TT (1111)	P1	P2
Glucose, mg/dL	126 ± 3	119 ± 3	112 ± 5	0.019	0.004	102 ± 3	99 ± 2	98 ± 1	0.401	0.223	118 ± 41	121 ± 43	116 ± 38	0.159	0.089
Total cholesterol, mg/dL	187 ± 2	189 ± 2	191 ± 4	0.603	0.456	191 ± 7	195 ± 3	193 ± 3	0.839	0.887	216 ± 38	217 ± 38	218 ± 40	0.370	0.722
LDL-C, mg/dL	110 ± 2	111 ± 2	113 ± 3	0.651	0.518	123 ± 6	123 ± 3	122 ± 2	0.919	0.963	133 ± 34	133 ± 34	133 ± 36	0.806	0.781
HDL-C, mg/dL	46.7 ± 0.7	47.6 ± 0.6	45.6 ± 1.0	0.236	0.305	50.4 ± 2.2	51.5 ± 1.1	51.3 ± 1.0	0.926	0.892	56.6 ± 13.8	57.2 ± 14.0	58.2 ± 15.3	0.014	0.014
Triglycerides, mg/dL	157 ± 6	156 ± 5	165 ± 9	0.686	0.890	117 ± 13	127 ± 7	132 ± 6	0.559	0.572	135 ± 80	134 ± 70	135 ± 80	0.512	0.866

	WHI African Americans					WHI Hispanic Americans				
	CC (453)	CT (304)	TT (50)	P1	P2	CC (147)	CT (214)	TT (74)	P1	P2
Glucose, mg/dL	108 ± 2	108 ± 2	103 ± 5	0.657	0.878	104 ± 2	101 ± 2	100 ± 3	0.493	0.731
Total cholesterol, mg/dL	223 ± 2	216 ± 2	218 ± 6	0.090	0.032	219 ± 3	221 ± 3	221 ± 4	0.888	0.495
LDL-C, mg/dL	141 ± 2	134 ± 2	135 ± 6	0.075	0.058	132 ± 3	133 ± 2	133 ± 4	0.934	0.514
HDL-C, mg/dL	58.1 ± 0.7	58.5 ± 0.8	59.9 ± 2.1	0.699	0.761	55.1 ± 1.1	54.8 ± 0.9	55.4 ± 1.6	0.948	0.847
Triglycerides, mg/dL	122 ± 3	119 ± 3	116 ± 8	0.527	0.290	162 ± 6	165 ± 5	156 ± 9	0.793	0.955

*: Values are means ± Standard Error of Mean. The rs3754686 SNP was determined in PREDIMED and imputed in BPRHS. The proxy rs309180 was genotyped in GOLDN and WHI studies.

**: General Linear Regression models with multivariable adjustment for the indicated covariates were fitted for each population.

1: P adjusted by sex, age, field center or race.

2: P adjusted for sex, age, field center or ancestry (BPRHS, WHI), family (GOLDN), BMI, smoking, drinking, physical activity, diabetes, medication and total energy intake.

In PREDIMED, some variables (glucose, LDL-C, HDL-C and triglycerides) included missing data point. Biochemical data were available for fasting glucose (n = 3886 participants) total cholesterol (n = 3964 participants), HDL cholesterol (n = 3912 participants), LDL cholesterol (n = 3883 participants), and triglycerides (n = 3935 participants).

Supplemental table S7. Associations of milk intake with fasting glucose and lipids in the whole population and stratified by sex or race in BPRHS, GOLDN, PREDIMED and WHI studies

	BPRHS			GOLDN			PREDIMED			WHI		
	Beta	SE	P*	Beta	SE	P*	Beta	SE	P*	Beta	SE	P*
Whole population												
Glucose, mg/dL	0.128	0.521	0.806 (0.730)	0.159	0.157	0.314 (0.823)	0.300	0.200	0.113 (0.136)	0.650	0.617	0.292 (0.590)
Total cholesterol, mg/dL	-0.387	0.460	0.400 (0.503)	-0.677	0.361	0.061 (0.034)	-0.054	0.023	0.828 (0.596)	0.406	0.938	0.665 (0.312)
LDL-C, mg/dL	-0.436	0.380	0.252 (0.432)	-0.505	0.300	0.093 (0.140)	0.106	0.211	0.613 (0.749)	0.509	0.892	0.569 (0.486)
HDL-C, mg/dL	-0.045	0.131	0.734 (0.639)	-0.046	0.117	0.697 (0.250)	-0.059	0.082	0.474 (0.364)	-0.282	0.319	0.377 (0.371)
Triglycerides, mg/dL	0.003	0.006	0.660 (0.730)	-0.002	0.002	0.434 (0.589)	-0.547	0.470	0.228 (0.251)	0.000	0.009	0.977 (0.628)
Men or WHI African-American women												
Glucose, mg/dL	0.279	1.016	0.784 (0.902)	0.228	0.241	0.344 (0.754)	0.711	0.352	0.044 (0.058)	1.051	0.733	0.152 (0.353)
Total cholesterol, mg/dL	-0.592	0.869	0.497 (0.903)	-0.950	0.475	0.046 (0.377)	0.181	0.370	0.622 (0.710)	0.295	1.155	0.798 (0.268)
LDL-C, mg/dL	-0.973	0.705	0.168 (0.847)	-0.797	0.391	0.042 (0.891)	0.622	0.329	0.059 (0.233)	0.360	1.092	0.742 (0.370)
HDL-C, mg/dL	-0.116	0.232	0.617 (0.931)	0.170	0.127	0.183 (0.049)	-0.240	0.121	0.049 (0.024)	-0.163	0.387	0.673 (0.511)
Triglycerides, mg/dL	0.016	0.012	0.178 (0.412)	-0.006	0.003	0.082 (0.249)	-0.490	0.790	0.537 (0.542)	-0.001	0.011	0.949 (0.769)
Women or WHI Hispanic-American women												
Glucose, mg/dL	0.074	0.613	0.905 (0.639)	0.098	0.201	0.628 (0.894)	0.127	0.270	0.643 (0.750)	-0.734	1.154	0.526 (0.412)
Total cholesterol, mg/dL	-0.288	0.544	0.596 (0.338)	-0.429	0.558	0.443 (0.033)	-0.223	0.303	0.462 (0.496)	0.449	1.613	0.781 (0.849)
LDL-C, mg/dL	-0.209	0.455	0.646 (0.086)	-0.210	0.473	0.657 (0.029)	-0.267	0.275	0.330 (0.395)	0.887	1.536	0.565 (0.794)
HDL-C, mg/dL	-0.002	0.161	0.992 (0.368)	-0.329	0.204	0.107 (0.983)	0.064	0.112	0.565 (0.545)	-0.472	0.572	0.411 (0.627)
Triglycerides, mg/dL	-0.004	0.007	0.595 (0.073)	0.003	0.004	0.347 (0.239)	-0.515	0.576	0.371 (0.393)	-0.007	0.019	0.704 (0.390)

*: *P* adjusted by sex, age, field center or ancestry (BPRHS, WHI), family (GOLDN), BMI, smoking, drinking, physical activity, diabetes, medication and total energy intake.

Beta indicates the increase/decrease of the CVD risk factor in mg/dL per 100 g/d of milk consumed. SE is expressed in the same units too.

BPRHS (n = 1232); GOLDN (n = 817); PREDIMED (fasting glucose (n = 3886 participants) total cholesterol (n = 3964 participants), HDL cholesterol (n = 3912 participants), LDL cholesterol (n = 3883 participants), and triglycerides (n = 3935 participants)); WHI (n = 1242).

Table S8. Incidence and hazard ratios (HR) for CVD depending on the MCM6-rs3754686 polymorphism after 4.8 years of median follow-up and stratified by sex

	Cases	Non-cases	person-y	Incidence	Model 1 HR	Model 1 95% CI	Model 1 P-value	Model 2 HR	Model 2 95% CI	Model 2 P-value	Model 3 P-value	Model 4 P-value
**Men n = 3,065**												
*MCM6 genotypes***												
TT	34	803	3664	9.3	1.00	(reference)		1.00	(reference)			
CT	86	1418	6425	13.4	1.49	(1.01-2.23)	0.049	1.50	(1.01-2.26)	0.049	0.068	0.064
CC	37	687	3093	12.0	1.32	(0.83-2.16)	0.246	1.33	(0.82-2.16)	0.246	0.239	0.237
TT (ref.)***					1.00	(reference)		1.00	(reference)			
(CC + TC) vs TT					1.44	(0.98-2.11)	0.063	1.45	(0.95-2.14)	0.064	0.079	0.075
Per variant allele (T)****					0.88	(0.70-1.09)	0.234	0.88	(0.70-1.09)	0.243	0.236	0.234
**Women n = 4,120**												
*MCM6 genotypes***												
TT	40	1105	4948	8.1	1.00	(reference)		1.00	(reference)			
CT	50	1992	8936	5.6	0.67	(0.45-1.03)	0.069	0.66	(0.43-1.01)	0.054	0.055	0.060
CC	20	913	3922	5.1	0.63	(0.36-1.08)	0.092	0.65	(0.38-1.13)	0.131	0.130	0.154
TT (ref.)***					1.00	(reference)		1.00	(reference)			
(CC + CT) vs TT					0.66	(0.45-0.98)	0.040	0.66	(0.45-0.98)	0.039	0.039	0.046
Per variant allele (T)****					1.30	(0.99-1.71)	0.062	1.28	(0.97-1.69)	0.083	0.082	0.098

P[§]-interaction sex*MCM6 polymorphism: 0.005

*: Crude incidence rates were expressed per 1000 person-years of follow-up.
: Codominant model. *: Recesive model.****: Additive model.
We used multivariable Cox regression models with length of follow-up as the primary time variable. Separate models were fitted for CVD and total mortality to estimate the corresponding HRs depending on the model.
Model 1: Adjusted for sex, age, field center and dietary intervention group.
Model 2: Model 1 adjusted for variables in model 1 plus BMI, diabetes, drinking, smoking, physical activity, medication (hypertension, dyslipemia and glucose) and total energy intake at baseline.
Model 3: Model 2 adjusted for variables in model 2 plus total milk intake.
Model 4: Model 3 additionally adjusted for total fat and carbohydrates at baseline.
[§]: P-value for interaction sex*MCM6 polymorphism in determining CVD incidence, obtained in Model 2. Further adjustments did not change the statistical significance.

Table S9. Incidence and hazard ratios (HR) for total mortality depending on the MCM6-rs3754686 polymorphism after 4.8 years of median follow-up and stratified by sex

					Model 1			Model 2			Model 3	Model 4
	Deaths	Non-cases	person-y	Incidence rate*	HR	95% CI	P-value	HR	95% CI	P-value	P-value	P-value
**Men n = 3,065**												
*MCM6 genotypes***												
TT	58	779	3669	15.8	1.00	(reference)		1.00	(reference)			
CT	83	1421	6433	12.9	0.83	(0.59-1.16)	0.280	0.84	(0.60-1.19)	0.325	0.324	0.411
CC	55	669	3101	17.7	1.10	(0.76-1.60)	0.615	1.09	(0.75-1.60)	0.647	0.647	0.511
TT (ref.)***					1.00	(reference)		1.00	(reference)			
(CC + TC) vs TT					0.92	(0.67-1.25)	0.594	0.92	(0.67-1.27)	0.626	0.624	0.793
Per variant allele (T)****					0.96	(0.79-1.16)	0.650	0.96	(0.78-1.17)	0.667	0.668	0.466
**Women n = 4,120**					Model 1			Model 2			Model 3	Model 4
*MCM6 genotypes***												
TT	46	1099	4949	9.3	1.00	(reference)		1.00	(reference)			
CT	56	1986	8944	6.3	0.65	(0.43-0.96)	0.029	0.62	(0.42-0.93)	0.020	0.029	0.021
CC	23	910	3922	5.9	0.60	(0.36-0.99)	0.049	0.64	(0.39-1.07)	0.089	0.084	0.072
TT (ref.)***					1.00	(reference)		1.00	(reference)			
(CC + TC) vs TT					0.63	(0.44-0.91)	0.014	0.63	(0.43-0.91)	0.014	0.014	0.013
Per variant allele (T)****					1.33	(1.03-1.72)	0.029	1.30	(1.01-1.70)	0.049	0.046	0.040

P§-interaction sex*MCM6 polymorphism: 0.032

*: Crude incidence rates were expressed per 1000 person-years of follow-up.

: Codominant model. *: Recesive model.****: Additive model.

We used multivariable Cox regression models with length of follow-up as the primary time variable. Separate models were fitted for CVD and total mortality to estimate the corresponding HRs depending on the model.

Model 1: Adjusted for sex, age, field center and dietary intervention group.

Model 2: Model 1 adjusted for variables in model 1 plus BMI, diabetes, drinking, smoking, physical activity, medication (hypertension, dyslipemia and glucose) and total energy intake at baseline.

Model 3: Model 2 adjusted for variables in model 2 plus total milk intake.

Model 4: Model 3 additionally adjusted for total fat and carbohydrates at baseline.

§: P-value for interaction sex*MCM6 polymorphism in determining mortality, obtained in Model 2. Further adjustments did not change the statistical significance.

Supplemental table S10. Incidence and hazard ratios (HR) for CVD depending on the MCM6-rs3754686 polymorphism after 4.8 years of median follow-up for the Mediterranean Diet intervention group and stratified by sex

	Cases	Non-cases	person-y	Incidence rate*	Model 1			Model 2			Model 3	Model 4
					HR	95% CI	P-value	HR	95% CI	P-value	P-value	P-value
**Total (men + women) n = 4,845**												
*MCM6 genotypes***												
TT	50	1310	6076	8.2	1.00	(reference)		1.00	(reference)			
CT	79	2282	10636	7.4	0.93	(0.65-1.33)	0.681	0.90	(0.63-1.29)	0.564	0.563	0.491
CC	36	1088	4944	7.3	0.89	(0.58-1.38)	0.612	0.86	(0.55-1.32)	0.482	0.480	0.508
TT (ref.)***					1.00	(reference)		1.00	(reference)			
(CC + TC) vs TT					0.92	(0.66-1.28)	0.614	0.89	(0.63-1.24)	0.481	0.480	0.441
Per variant allele (T)****					1.06	(0.85-1.32)	0.602	1.08	(0.87-1.35)	0.470	0.469	0.487
**Men n = 2,119**					Model 1			Model 2			Model 3	Model 4
*MCM6 genotypes***												
TT	21	568	2661	7.9	1.00	(reference)		1.00	(reference)			
CT	51	961	4515	11.3	1.49	(0.89-2.49)	0.127	1.47	(0.88-2.47)	0.145	0.149	0.159
CC	26	492	2277	11.4	1.47	(0.82-2.63)	0.198	1.38	(0.77-2.48)	0.281	0.286	0.276
TT (ref.)***					1.00	(reference)		1.00	(reference)			
(CC + CT) vs TT					1.48	(0.91-2.41)	0.114	1.44	(0.88-2.36)	0.148	0.152	0.180
Per variant allele (T)****					0.84	(0.63-1.10)	0.201	0.86	(0.65-1.14)	0.290	0.299	0.286
**Women n = 2,726**					Model 1			Model 2			Model 3	Model 4
*MCM6 genotypes***												
TT	29	742	3416	8.5	1.00	(reference)		1.00	(reference)			
CT	28	1321	6122	4.6	0.53	(0.32-0.90)	0.019	0.54	(0.32-0.92)	0.024	0.024	0.028
CC	10	596	2668	3.7	0.48	(0.23-0.98)	0.046	0.51	(0.25-1.06)	0.069	0.070	0.083
TT (ref.)***					1.00	(reference)		1.00	(reference)			
(CC + CT) vs TT					0.52	(0.30-0.84)	0.008	0.54	(0.33-0.88)	0.013	0.013	0.016
Per variant allele (T)****					1.55	(1.08-2.22)	0.017	1.50	(1.05-2.17)	0.028	0.028	0.034

*: Crude incidence rates were expressed per 1000 person-years of follow-up.
: Codominant model. *: Recesive model.****: Additive model.
We used multivariable Cox regression models with length of follow-up as the primary time variable. Separate models were fitted for CVD and total mortality to estimate the corresponding HRs depending on the model.
Model 1: Adjusted for sex, age, field center and dietary intervention group.
Model 2: Model 1 adjusted for variables in model 1 plus BMI, diabetes, drinking, smoking, physical activity, medication (hypertension, dyslipemia and glucose) and total energy intake at baseline.
Model 3: Model 2 adjusted for variables in model 2 plus total milk intake.
Model 4: Model 3 additionally adjusted for total fat and carbohydrates at baseline.

Supplemental table S11. Incidence and hazard ratios (HR) for CVD depending on the MCM6-rs3754686 polymorphism after 4.8 years of median follow-up for the Control group and stratified by sex

	Cases	Non-cases	person-y	Incidence rate*	Model 1 HR	Model 1 95% CI	Model 1 P-value	Model 2 HR	Model 2 95% CI	Model 2 P-value	Model 3 P-value	Model 4 P-value
**Total (men + women) n = 2,340**												
*MCM6 genotypes***												
TT	24	598	2535	9.5	1.00	(reference)		1.00	(reference)			
CT	57	1128	4726	12.1	1.29	(0.78-2.08)	0.329	1.02	(0.77-1.36)	0.890	0.325	0.325
CC	21	512	2071	10.1	1.13	(0.62-2.05)	0.694	0.95	(0.69-1.35)	0.764	0.667	0.628
TT (ref.)***					1.00	(reference)		1.00	(reference)			
(CC + TC) vs TT					1.21	(0.77-1.93)	0.410	1.23	(0.77-1.98)	0.383	0.373	0.362
Per variant allele (T)****					0.97	(0.73-1.28)	0.830	0.94	(0.71-1.25)	0.658	0.631	0.592
**Men n = 946**												
*MCM6 genotypes***												
TT	13	235	1003	13.0	1.00	(reference)		1.00	(reference)			
CT	35	457	1910	18.3	1.47	(0.78-2.79)	0.238	1.53	(0.79-2.97)	0.209	0.198	0.221
CC	11	195	816	13.5	1.02	(0.45-2.29)	0.962	1.15	(0.50-2.66)	0.734	0.711	0.722
TT (ref.)***					1.00	(reference)		1.00	(reference)			
(CC + CT) vs TT					1.33	(0.72-2.48)	0.365	1.42	(0.75-2.70)	0.284	0.269	0.293
Per variant allele (T)****					0.98	(0.68-1.41)	0.891	0.92	(0.63-1.34)	0.672	0.644	0.660
**Women n = 1,394**												
*MCM6 genotypes***												
TT	11	363	1532	7.2	1.00	(reference)		1.00	(reference)			
CT	22	671	2816	7.8	1.03	(0.50-2.15)	0.934	0.99	(0.47-2.10)	0.990	0.989	0.973
CC	10	317	1255	8.0	1.03	(0.43-2.48)	0.950	1.09	(0.45-2.67)	0.843	0.841	0.919
TT (ref.)***					1.00	(reference)		1.00	(reference)			
(CC + CT) vs TT					1.03	(0.51-2.07)	0.933	1.02	(0.50-2.08)	0.949	0.949	0.872
Per variant allele (T)****					0.99	(0.64-1.53)	0.950	0.96	(0.61-1.50)	0.849	0.848	0.820

*: Crude incidence rates were expressed per 1000 person-years of follow-up.
: Codominant model. *: Recesive model.****: Additive model.
We used multivariable Cox regression models with length of follow-up as the primary time variable. Separate models were fitted for CVD and total mortality to estimate the corresponding HRs depending on the model.
Model 1: Adjusted for sex, age, field center and dietary intervention group.
Model 2: Model 1 adjusted for variables in model 1 plus BMI, diabetes, drinking, smoking, physical activity, medication (hypertension, dyslipemia and glucose) and total energy intake at baseline.
Model 3: Model 2 adjusted for variables in model 2 plus total milk intake.
Model 4: Model 3 additionally adjusted for total fat and carbohydrates at baseline.

Supplemental table S12. Incidence and hazard ratios (HR) for total mortality depending on the MCM6-rs3754686 polymorphism after 4.8 years of median follow-up for the Mediterranean Diet intervention group and stratified by sex

	Total (men + women) n = 4,845											
					Model 1			Model 2			Model 3	Model 4
	Deaths	Non-cases	person-y	Incidence rate*	HR	95% CI	P-value	HR	95% CI	P-value	P-value	P-value
*MCM6 genotypes***												
TT	78	1282	6079	12.8	1.00	(reference)		1.00	(reference)			
CT	90	2271	10648	8.5	0.67	(0.49-0.90)	0.009	0.65	(0.48-0.89)	0.007	0.007	0.007
CC	53	1071	4957	10.7	0.82	(0.58-1.17)	0.269	0.79	(0.55-1.14)	0.210	0.206	0.261
TT (ref.)***					1.00	(reference)		1.00	(reference)			
(CC + TC) vs TT					0.72	(0.54-0.95)	0.018	0.70	(0.52-0.93)	0.012	0.012	0.015
Per variant allele (T)****					1.14	(0.94-1.37)	0.181	0.93	(0.70-1.24)	0.611	0.140	0.175
	**Men n = 2,119**											
					Model 1			Model 2			Model 3	Model 4
*MCM6 genotypes***												
TT	43	546	2662	16.2	1.00	(reference)		1.00	(reference)			
CT	51	961	4514	11.3	0.73	(0.49-1.10)	0.132	0.74	(0.49-1.20)	0.153	0.154	0.196
CC	40	478	2284	17.5	1.09	(0.70-1.69)	0.705	1.02	(0.65-1.59)	0.946	0.943	0.751
TT (ref.)***					1.00	(reference)		1.00	(reference)			
(CC + TC) vs TT					0.85	(0.59-1.23)	0.390	0.83	(0.57-1.22)	0.347	0.348	0.451
Per variant allele (T)****					0.96	(0.76-1.22)	0.752	1.00	(0.78-1.26)	0.965	0.962	0.967
	**Women n = 2,726**											
					Model 1			Model 2			Model 3	Model 4
*MCM6 genotypes***												
TT	35	736	3416	10.2	1.00	(reference)		1.00	(reference)			
CT	39	1310	6124	6.4	0.60	(0.38-0.94)	0.027	0.56	(0.37-0.94)	0.026	0.029	0.030
CC	13	593	2666	4.9	0.47	(0.25-0.90)	0.022	0.53	(0.28-1.02)	0.056	0.053	0.046
TT (ref.)***					1.00	(reference)		1.00	(reference)			
(CC + TC) vs TT					0.56	(0.36-0.86)	0.008	0.57	(0.37-0.89)	0.013	0.013	0.013
Per variant allele (T)****					1.51	(1.10-2.07)	0.010	1.45	(1.05-2.00)	0.025	0.024	0.021

*: Crude incidence rates were expressed per 1000 person-years of follow-up.
: Codominant model. *: Recesive model.****: Additive model.
We used multivariable Cox regression models with length of follow-up as the primary time variable. Separate models were fitted for CVD and total mortality to estimate the corresponding HRs depending on the model.
Model 1: Adjusted for sex, age, field center and dietary intervention group.
Model 2: Model 1 adjusted for variables in model 1 plus BMI, diabetes, drinking, smoking, physical activity, medication (hypertension, dyslipemia and glucose) and total energy intake at baseline.
Model 3: Model 2 adjusted for variables in model 2 plus total milk intake.
Model 4: Model 3 additionally adjusted for total fat and carbohydrates at baseline.

Supplemental table S13. Incidence and hazard ratios (HR) for total mortality depending on the MCM6-rs3754686 polymorphism after 4.8 years of median follow-up for the Control group and stratified by sex

	Deaths	Non-cases	person-y	Incidence rate*	Model 1 HR	Model 1 95% CI	Model 1 P-value	Model 2 HR	Model 2 95% CI	Model 2 P-value	Model 3 P-value	Model 4 P-value
**Total (men + women) n = 2,340**												
*MCM6 genotypes***												
TT	26	596	2538	10.2	1.00	(reference)		1.00	(reference)			
CT	49	1136	4740	10.3	1.00	(0.62-1.62)	0.988	0.98	(0.60-1.60)	0.931	0.928	0.895
CC	25	508	2073	12.1	1.12	(0.64-1.96)	0.686	1.16	(0.66-2.05)	0.601	0.615	0.612
TT (ref.)***					1.00	(reference)		1.00	(reference)			
(CC + TC) vs TT					1.04	(0.66-1.63)	0.865	1.03	(0.65-1.64)	0.889	0.898	0.921
Per variant allele (T)****					0.94	(0.71-1.25)	0.690	1.15	(0.95-1.40)	0.143	0.543	0.624
**Men n = 946**												
*MCM6 genotypes***												
TT	15	233	1007	14.9	1.00	(reference)		1.00	(reference)			
CT	32	460	1919	16.7	1.11	(0.60-2.06)	0.736	1.06	(0.56-2.02)	0.854	0.879	0.852
CC	15	191	816	18.4	1.21	(0.54-2.49)	0.614	1.33	(0.63-2.82)	0.452	0.454	0.470
TT (ref.)***					1.00	(reference)		1.00	(reference)			
(CC + TC) vs TT					1.14	(0.63-2.05)	0.662	1.14	(0.62-2.09)	0.685	0.703	0.690
Per variant allele (T)****					0.91	(0.64-1.31)	0.613	0.87	(0.59-1.27)	0.457	0.460	0.474
**Women n = 1,394**												
*MCM6 genotypes***												
TT	11	363	1533	7.2	1.00	(reference)		1.00	(reference)			
CT	17	676	2821	6.0	0.90	(0.42-1.93)	0.780	0.76	(0.35-1.66)	0.487	0.476	0.462
CC	10	317	1256	8.0	1.12	(0.47-2.69)	0.796	0.94	(0.39-2.29)	0.889	0.922	0.898
TT (ref.)***					1.00	(reference)		1.00	(reference)			
(CC + TC) vs TT					0.97	(0.48-1.97)	0.928	0.82	(0.40-1.68)	0.581	0.583	0.806
Per variant allele (T)****					0.95	(0.60-1.49)	0.817	1.04	(0.65-1.66)	0.870	0.898	0.877

*: Crude incidence rates were expressed per 1000 person-years of follow-up.
: Codominant model. *: Recesive model.****: Additive model.
We used multivariable Cox regression models with length of follow-up as the primary time variable. Separate models were fitted for CVD and total mortality to estimate the corresponding HRs depending on the model.
Model 1: Adjusted for sex, age, field center and dietary intervention group.
Model 2: Model 1 adjusted for variables in model 1 plus BMI, diabetes, drinking, smoking, physical activity, medication (hypertension, dyslipemia and glucose) and total energy intake at baseline.
Model 3: Model 2 adjusted for variables in model 2 plus total milk intake.
Model 4: Model 3 additionally adjusted for total fat and carbohydrates at baseline.

Supplemental table S14. Incidence and hazard ratios (HR) for CVD incidence depending on milk intake and stratified by sex

	**Total (men + women) n = 7,127**										
					Model 1			Model 2			Model 3
Total Milk Intake	Cases	Non-cases	person-y	Incidence rate*	HR	95% CI	P-value	HR	95% CI	P-value	P-value
Proximate tertiles											
< 200 g/d	64	1612	7073	9.0	1.00	(reference)		1.00	(reference)		
200 g/d	117	3136	14151	8.3	0.88	(0.65-1.19)	0.407	0.86	(0.63-1.17)	0.346	0.279
> 200 g/d	85	2113	9561	8.9	1.08	(0.74-1.41)	0.937	0.93	(0.66-1.31)	0.686	0.207
Dichotomous											
≤ 200 g/d					1.00	(reference)		1.00	(reference)		
> 200 g/d					1.08	(0.74-1.41)	0.937	1.03	(0.78-1.36)	0.829	0.705
P§-interaction sex*milk (dichotomous): 0.687											
	**Men n = 3,041**										
					Model 1			Model 2			Model 3
Proximate tertiles											
< 200 g/d	44	807	3642	12.1	1.00	(reference)		1.00	(reference)		
200 g/d	68	1346	6094	11.2	0.86	(0.57-1.23)	0.356	0.83	(0.56-1.22)	0.332	0.239
> 200 g/d	44	732	3352	13.1	0.98	(0.64-1.50)	0.912	0.96	(0.62-1.49)	0.854	0.542
Dichotomous											
≤ 200 g/d					1.00	(reference)		1.00	(reference)		
> 200 g/d					1.09	(0.76-1.57)	0.627	1.08	(0.75-1.58)	0.671	0.946
	**Women n = 4,086**										
					Model 1			Model 2			Model 3
Proximate tertiles											
< 200 g/d	20	805	3432	5.8	1.00	(reference)		1.00	(reference)		
200 g/d	49	1790	8055	6.1	1.00	(0.59-1.68)	0.985	0.96	(0.57-1.63)	0.892	0.732
> 200 g/d	41	1381	6208	6.6	1.09	(0.63-1.86)	0.765	0.89	(0.51-1.55)	0.682	0.275
Dichotomous											
≤ 200 g/d					1.00	(reference)		1.00	(reference)		
> 200 g/d					1.09	(0.74-1.61)	0.669	0.91	(0.61-1.35)	0.664	0.240

*: Crude incidence rates were expressed per 1000 person-years of follow-up.

: Codominant model. *: Recesive model.****: Additive model.

We used multivariable Cox regression models with length of follow-up as the primary time variable. Separate models were fitted for CVD and total mortality to estimate the corresponding HRs depending on the model.

Model 1: Adjusted for sex, age, field center and dietary intervention group.

Model 2: Model 1 adjusted for variables in model 1 plus BMI, diabetes, drinking, smoking, physical activity, medication (hypertension, dyslipemia and glucose) and total energy intake at baseline.

Model 3: Model 2 additionally adjusted for total fat and carbohydrates at baseline.

§: P-value for interaction sex*milk (dichotomous) in determining CVD incidence obtained in Model 2. Further adjustment did not change the statistical significance.

Table S15. Description, design and protocols of the BPRHS, GOLDN, PREDIMED and WHI studies

Study:	Boston Puerto Rican Health Study	BPRHS
Website URL	https://www.uml.edu/Research/CPHHD/	
ClinicalTrials.gov Identifier	NCT01231958	

Study History and Recruitment	Age/sex
From June 2004 to October 2009, self-identified Puerto Ricans, aged 45-75 years and residing in the Boston, MA, USA metro area, were recruited through door-to-door enumeration and community approaches. Of those invited, 1,811 (86.5%) agreed to be interviewed.	Men (30%): age (yrs) 56.7±8.0<br>Women (70%): age(yrs) 57.4±7.7

Health Data Collection	Genotyping
Weight was measured using a clinical scale (Toledo Weight Plate, Model I5S, Bay State and Systems Inc. Burlington, MA). Height was measured with a SECA 214 Portable Stadiometer. Knee height was measured to estimate standing height for participants who were unable to stand. Standing height, knee height, weight, and waist and hip circumferences were measured in duplicate. Other health information was obtained through questionnaires, administered by bilingual interviewers.	In the BPRHS, the MCM6-rs3754686 was imputed. For imputation, the 1000 genome project genotypes were used with reference haplotype panels from the Nov.23 2010 release of the 1000 Genomes project using a MaCH-Admix (http://www.unc.edu/~yunmli/MaCH-Admix/) by Yun Li (University of North Carolina of Chapel Hill). The BPRHS dense genotyping data for imputation were obtained using the Affymetrix's Axiom Genome-Wide LAT Array (717,275 autosomal SNPs genotyped). These data were selected to create the input file for MaCH-Admix based on the following quality control criteria: call rate ≥97%, Hardy-Weinberg P-value≥10^{-6}, and MAF≥0.05.

Blood sample collection and handling
Blood was collected after a 12 hour fast and was drawn in the home by a certified phlebotomist on the morning following the home interview. A portable centrifuge was used in the home to immediately spin down the blood samples. Blood samples were carried back to the Nutrition Evaluation Laboratory (NEL) at the Human Nutrition Research Center on Aging on the day of collection in coolers equipped with dry ice. Blood samples were immediately cooled to 4°C and the plasma separated within 4 hours in a refrigerated centrifuge. Samples were expected to be online for testing within 1 hour from arrival into laboratory, and were kept cold until analyzed.

Laboratory Quality Control

Biochemical measurements were measured in a state and federal licensed laboratory according to standard operating procedures. Control of pre-analytical variation was maximized through adherence to a manual of operations. Glucose was measured with intra- and inter-assay C.V.s of 2.0% and 3.2% respectively. Total cholesterol was measured by an enzymatic procedure with intra- and inter-assay CVs of 2.0% and 2.8% respectively. Triglycerides were measured by a series of coupled enzymatic reactions with intra- and inter-assay CVs of 2.0% and 3.4% respectively. HDL was measured by a two-phase reaction with colorimetric endpoint detection, with intra- and inter-assay CVs of 3.0% and 5.0% respectively.

Study:	Genetics of Lipid Lowering Drugs and Diet Network	GOLDN
Website URL	https://dsgweb.wustl.edu/goldn/	
ClinicalTrials.gov Identifier	NCT00083369	

Study History and Recruitment	Age/sex
GOLDN was initiated in 2002. Participants were re-recruited from 3-generational pedigrees from two NHLBI Family Heart Study field centers (Minneapolis, Minneapolis, and Salt Lake City, Utah). Nearly all individuals were of European ancestry.	Men (49%): age (yrs) 49±16<br>Women (51%) : age(yrs) 49±16

Health Data Collection	Genotyping
Weight was taken with minimal clothing on a balance. Results were recorded to the nearest pound, rounding down. Height was measured while standing as straight as possible without shoes with feet flat on the floor. Height was recorded to the nearest centimeter, rounding down to the nearest centimeter. Clinical and lifestyle questionnaires were administered by a trained interviewer.	For the GOLDN study, genotypes were obtained using the genome-wide Human SNP Array 6.0 (Affymetrix, Santa Clara, CA, USA, www.affymetrix.com). In GOLDN, the proxy SNP MCM6-rs309180, with a high LD with the MCM6-rs3754686 (D'=1 and r^2=0.95), was genotyped.

Blood sample collection and handling
Blood was collected after a 12 hour overnight fast. Specimens were required to be non-hemolyzed. Blood from visits 1-4 were stored at -70 degrees C at field centers until all visits were complete, and then analysis was performed. For the current study, analyses were performed with blood obtained at visit 2. Blood for glucose and lipids was stored in yellow cap tubes.

Laboratory Quality Control
Quality control procedures were performed quarterly by the Collaborative Studies Clinical Laboratory (CSCL) Minneapolis, Minnesota in conjunction with the CDC. Lipid proficiency specimens (n=36) were distributed by the CDC and assayed by the CSCL in batches at weekly intervals. Acceptable accuracy limits were as follows: total cholesterol: CDC mean ± 3%, triglycerides: <200 = CDC mean ± 8 mg/dL; triglycerides >200= CDC mean ± 5%, HDL-C: CDC mean ± 5%

Study:	PREVENCIÓN CON DIETA MEDITERRÁNEA	PREDIMED
Website URL	http://www.predimed.es/	
Controlled-Trials.com number	ISRCTN35739639	

Study History and Recruitment	Age/sex
The PREDIMED trial (*Prevención con Dieta Mediterránea*) was a parallel-group, multicenter, randomized trial. The trial was designed and conducted by the authors, and the protocol was approved by the institutional review boards at all study locations.<br><br>Beginning on October 1, 2003, participants were randomly assigned, in a 1:1:1 ratio, to one of three dietary intervention groups: a Mediterranean diet supplemented with extra-virgin olive oil, a Mediterranean diet supplemented with nuts, or a control diet. Randomization was performed centrally by means of a computer-generated random-number sequence. From October 2003 through June 2009, a total of 8713 candidates were screened for eligibility, and 7447 were randomly assigned to one of the three study groups.	Eligible participants were men (55 to 80 years of age) and women (60 to 80 years of age).

Health Data Collection	Genotyping
Weight, height, and waist circumference were directly measured. A general medical questionnaire, a 137-item validated food-frequency questionnaire, and the Minnesota Leisure-Time Physical Activity Questionnaire were administered on a yearly basis. Information from the food-frequency questionnaire was used to calculate intake of energy and nutrients.<br><br>The primary end point was a composite of myocardial infarction, stroke, and death from cardiovascular causes. Secondary end points were stroke, myocardial infarction, death from cardiovascular causes, and death from any cause. We used four sources of information to identify end points: repeated contacts with participants, contacts with family physicians, a yearly review of medical records, and consultation of the National Death Index. All medical records related to end points were examined by the end-point adjudication committee, whose members were unaware of the study-group assignments. Only end points that were confirmed by the adjudication committee and that occurred between October 1, 2003, and December 1, 2010, were included in the analyses.	Genomic DNA was extracted from buffy coat. We genotyped the MCM6-rs3754686 polymorphism in all PREDIMED participants with DNA available on a 7900HT Sequence Detection System (Applied Biosystems) by using a fluorescent allelic discrimination TaqMan assay.

Blood sample collection and handling

Blood samples were obtained after an overnight fast and were frozen at −80°C. Fasting glucose, total cholesterol, triglycerides, HDL cholesterol, and LDL cholesterol were measured by using standard enzymatic methods. In participants whose triglyceride levels were <400 mg/dL, LDL cholesterol concentrations were estimated by using the Friedewald formula. Biochemical measures were available for nearly 7000 participants at baseline.

Laboratory Quality Control

Biochemical analysis was carried out in regional and national licensed laboratories according to standard operating procedures. Control of pre-analytical variation was maximized through adherence to a manual of operations. Glucose was measured with intra- and inter-assay C.V.s of 2.0% and 3.2% respectively. Total cholesterol was measured by an enzymatic procedure with intra- and inter-assay C.V.s of 2.0% and 2.8% respectively. Triglycerides were measured by a series of coupled enzymatic reactions with intra- and inter-assay C.V.s of 2.0% and 3.4% respectively. HDL was measured by a two-phase reaction with colorimetric endpoint detection, with intra- and inter-assay C.V.s of 3.0% and 5.0% respectively.

Study:	WOMEN’S HEALTH INITIATIVE	WHI
Website URL	https://www.whi.org	
ClinicalTrials.gov Identifier	NCT00000611	

Study History and Recruitment	Age/sex
WHI began in 1993 and is ongoing. Participants were recruited and enrolled at 40 clinical centers throughout the US. Recruitment methods included: mass mailings (primary method), community presentations, newspapers and articles, TV and radio, and health fairs. A total of 161,808 postmenopausal women aged 50–79 years old were recruited. Data for the current study were collected at baseline from the Observational Study, and were limited to African American and Hispanic women.	African American women (69%): age(yrs) 61.1±6.8<br>Hispanic American women (31%): age(yrs) 60.0±6.6

Health Data Collection	Genotyping
Health information relevant to the current study (such as weight and height) was collected at a clinic visit by trained clinical stuff. Other information, such as age and ethnicity were collected at baseline by self-report. At baseline and the first follow-up clinic visit, which occurred 3 years after baseline, Observational Study participants completed questionnaires on medical, lifestyle and psychosocial characteristics.	For the WHI SNP Health Association Resource (SHARe) study, genotypes were obtained using the genome-wide Human SNP Array 6.0 (Affymetrix, Santa Clara, CA, USA, www.affymetrix.com). In WHI, the proxy SNP MCM6-rs309180, with a high LD with the MCM6-rs3754686 (D’=1 and r^2>0.96), was genotyped.

Blood sample collection and handling
Blood was collected after a 12 hour fast and was maintained at 4 degrees C for up to 1 hour until plasma or serum was separated from cells. Centrifuged aliquots were stored in freezers (at -70 degrees C) within 2 h of collection and sent on dry ice to the central repository, where storage at -70 degrees C was maintained.

Laboratory Quality Control
The accuracy and precision of the lipid assays were regularly monitored with the CDC/NHLBI Lipid Standardization Program to control for any potential drift over time. The CDC/NHLBI Laboratory Quality Assurance and Standardization Program provides the clinical laboratory community with performance guidelines and equipment recommendations that meet the following standards: 1) cholesterol tests with bias from the reference method ≤ 3.0% and a coefficient of variation (CV) ≤ 3.0% 2) HDL cholesterol should be measured with a bias from the reference method ≤ 5% and methods perform with a CV ≤ 4% at ≥ 42 mg/dL (1.09mmol/L) and a standard deviation of ≤ 1.7 mg/dL (0.044 mmol/L) at < 42 mg/dL (1.09 mmol/L) and 3) LDL cholesterol with a bias from the reference method ≤ 4% and perform with a CV ≤ 4%.
